# Variability in ITS1 and ITS2 sequences of historic herbaria and extant (fresh) *Phalaris* species (Poaceae)

**DOI:** 10.1186/s12870-021-03284-z

**Published:** 2021-11-06

**Authors:** Allison L. Graper, Andrzej K. Noyszewski, Neil O. Anderson, Alan G. Smith

**Affiliations:** 1 Department of Horticultural Science, University of Minnesota, 1970 Folwell Avenue, Saint Paul, MN 55108 USA; 2 Present Address: Quality Control Analyst III, Aldevron, ND Fargo, USA

**Keywords:** Herbarium specimens, DNA barcoding, PCR amplification, Diagnostic SNPs

## Abstract

**Background:**

*Phalaris* species (*Poaceae*) occupy diverse environments throughout all continents except Antarctica. *Phalaris arundinacea* is an important forage, ornamental, wetland restoration and biofuel crop grown globally as well as being a wetland invasive. The nuclear ribosomal internal transcribed spacer (ITS) region has been used for *Phalaris* barcoding as a DNA region with high nucleotide diversity for *Phalaris* species identification. Recent findings that *P. arundinacea* populations in Minnesota USA are most likely native and not European prompted this analysis to determine whether Eurasian vs. native North American *P. arundinacea* differed in ITS regions. Our objectives were to amplify and compare ITS regions (ITS1 and ITS2) of historic herbaria (1882–2001) and extant (fresh) *Phalaris* specimens; analyze ITS regions for species-specific polymorphisms (diagnostic SNPs) and compare ITS regions of historic *Phalaris* specimens with known, extant *Phalaris* species.

**Results:**

We obtained complete ITS1 and ITS2 sequences from 31 *Phalaris* historic (herbaria samples, 1908 to 2001) and five extant (fresh) specimens*.* Herbaria *Phalaris* specimens did not produce new SNPs (single nucleotide polymorphisms) not present in extant specimens. Diagnostic SNPs were identified in 8/12 (66.6%) *Phalaris* species. This study demonstrates the use of herbaria tissue for barcoding as a means for improved species identification of *Phalaris* herbaria specimens. No significant correlation between specimen age and genomic DNA concentration was found. *Phalaris arundinacea* showed high SNP variation within its clade, with the North American being distinctly different than other USA and most Eurasian types, potentially allowing for future identification of specific SNPs to geographic origin.

**Conclusions:**

While not as efficient as extant specimens to obtain DNA, *Phalaris* herbaria specimens can produce high quality ITS sequences to evaluate historic genetic resources and facilitate identification of new species-specific barcodes. No correlation between DNA concentration and age of historic samples (119 year range) occurred. Considerable polymorphism was exhibited in the *P. arundinacea* clade with several N. American accessions being distinct from Eurasian types. Further development of within species- and genus-specific barcodes could contribute to designing PCR primers for efficient and accurate identification of N. American *P. arundinacea*. Our finding of misidentified *Phalaris* species indicates the need to exercise stringent quality control measures on newly generated sequence data and to approach public sequence databases in a critical way.

**Supplementary Information:**

The online version contains supplementary material available at 10.1186/s12870-021-03284-z.

## Background

From the 1980s onwards, sequence-based molecular phylogenetic studies in plants relied primarily on plastid genome spacers and genes, particularly *rbcL* (ribulose-bisphosphate carboxylase in the chloroplast genome) [1]. Risks of using such uniparentally inherited sequences for phylogenetics necessitated the development and implementation of nuclear markers to reflect biparental trait inheritance [2]. The internal transcribed spacer (ITS) region of the nuclear ribosomal cistron, 18S–5.8S-26S, initially used by Baldwin’s laboratory [3–5] quickly became the universally applied tool for molecular-based phylogenetic research for several reasons [6], not least of which included that 18S–26S rDNA arrays and their products are essential components of eukaryotic nucleolus organizing regions (NORs). Consequently, numerous plant families, genera and species have been analyzed for variance in ITS sequence differences, particularly for phylogenetic studies, species identification (barcoding) as well as ascertaining cultivar or genotype identities.

Such is the case with the genus *Phalaris* L. which is an important forage, ornamental, birdseed, wetland remediation/restoration and biofuel crop grown across the globe as well as being recognized as an invasive wetland species [7–9]. The genus *Phalaris* (Poaceae, grass family), classified in the Aveneae-Poeae section of the subfamily Pooideae, contains 20 species in the latest taxonomic treatises [10–15], although it previously included as many as 25 taxa. All *Phalaris* species in this monophyletic genus are cool-season grasses, with either annual or perennial life histories, from both the New and Old Worlds, varying in basic chromosome numbers of *x =* 6 and *x =* 7 and include a polyploid series from 2*x* to 8*x* [16–18]. Polyploid *Phalaris* exist in both Europe and North America, in fact polyploidy predominates throughout Europe and Asia. The species are widely adapted across the globe and are not restricted to one hemisphere [19]; six floret types distinguish the species as well as other diagnostic traits such as the presence of characteristic ligules [15]. The centers of origin and diversity are the Mediterranean Basin while a secondary center of diversity exists in western North America [7, 17–19]. The most cosmopolitan species is *P. arundinacea* L.*,* circumpolar in distribution in the northern hemisphere. Most likely, a diploid ancestor of *P. arundinacea* came across on the land bridge of the Bering Strait into the present-day State of Alaska, USA [20] during the late Tertiary period [21].

*Phalaris* has a lengthy taxonomic history with the earliest references arising in the first Century CE (common era), with *P. canariensis* being described by Dioscorides with an accompanying drawing from the Byzantine period (~ 525 CE) [22], although [7] argued that the drawing did not clearly delineate this species. Two species were given scientific names in quadrinomial nomenclature early in the 1600s: *P. major semine albo (*cf. *P. canariensis* L.*)* and *P. major semine nigro (*cf. *P. minor* Retz.), prior to the Linnaean era of binomial nomenclature [23, 24]*.*

Of particular importance within the genus, two species are of primary commercial value as cultivated crops, i.e. *P. arundinacea* L. (reed canarygrass; grown for ornamental, forage, biofuel and remediation/restoration efforts) [7–9] and *P. canariensis* L. (canarygrass; grown for birdseed) [25], although additional species are also cultivated: *P. aquatica, P. minor* [17, 18] and *P.* x*daviesii* [26]. Seed of both *P. arundinacea* and *P. canariensis* are commercially produced in Roseau, Minnesota USA [25], the state with the highest concentration and wetland surface area coverage (50–100% of wetland area) of native, yet invasive, *P. arundinacea* in the continental USA and in North America [27]. The species also is native to Eurasia [7, 28] and North America [29]. As a widely adaptable species, it is able to withstand a variety of conditions including frost, drought, partial shade and poorly drained soil [30]. While minor variation, i.e., plant height and biomass, exists due to genetic and environmental factors, both the native *P. arundinacea* North American types and those native to Eurasia are virtually indistinguishable for any morphological trait [31], since all possess ligules [7] and the same floret type, “Floret Type 4” [15], although the floret type cannot be used when collecting vegetative genotypes for analyses. The North American and Eurasian types (using both extant and historic or herbaria specimens) have been separated, however, using biochemical (allozymes) [28] and molecular markers, such as ISSRs (inter-simple sequence repeats) [32], AFLPs (amplified fragment polymorphisms) [33–35], SNPs (single nucleotide polymorphisms) [29, 36], as well as ITS regions [11, 15, 19, 20].

Herbarium specimens are museum-quality, pressed and dried plant samples deposited and preserved in global herbaria to serve in future research [37]. Plant specimens are typically mounted on acid-free paper with the primary goal of preserving specimen integrity and visual quality to allow for future systematic and taxonomic studies [37]. Herbaria contain an extensive collection of *Phalaris* species past germplasm (pre-1900), that were collected by early land surveyors during European settlement [38, 39]. Those herbarium specimens can serve as an informative resource for comparative genetics, genomics and systematics, to describe changes of *Phalaris* species germplasm over time. Less than optimal early specimen preservation methods can lead to various degrees of DNA degradation [29]. Estimation of the genetic distance between individuals and populations is based on single nucleotide polymorphism (SNP) and depends on the proper nucleotide base calling. Nucleotide misincorporations were found during amplification of ancient DNA and could cause false SNP recognition [40, 41]. False SNP recognition in herbaria specimens can be highly problematic when proper relatedness needs to be determined in comparison to fresh tissue collection. Here we compared SNPs found in genomic DNA obtained from herbaria specimens (collection years from 1882 to 2001) and fresh tissue (June 17th, 2019) to determine if amplification of DNA obtained from herbaria specimens resulted in varying SNP differences in comparison to DNA obtained from fresh tissue.

Plastid *trnT-F, trnL-F* and the nuclear ribosomal ITS region (ITS1–5.8S-ITS2) were amplified/sequenced in the core tribe Aveneae (oats) for taxonomic reconstruction of the Aveneae-Poeae-Seslerieae complex in the Poaceae [11]. That study included the genera *Anthoxanthum, Hierochloe* and *Phalaris,* which reside in the Phalarideae (sub Panicoideae). Alignment or clustering vs. bootstraps of ITS showed *Phalaris canariensis* and *P. truncata* to be very strongly supported (100/100; posterior probability support or PPS/bootstrap support) whereas *P. coerulescens* scored 100/95 in relation to these two *Phalaris* species [11]. The use of plastid and nuclear ribosomal markers provided highly accurate taxonomic delineation of the associated genera and species in the Aveneae-Poeae-Seslerieae complex wherein *Phalaris* was classified as a “small, less-diversified satellite lineage” [11]. Chloroplast DNA (13 intergenic sequence regions) and AFLPs were subsequently used to distinguish among North American and European *Phalaris arundinacea* herbaria samples [9]. Chloroplast markers supported AFLP findings that North American races of *P. arundinacea* were distinctly different from European types (which had higher genetic diversity) plus the added finding of a separate Scandinavian chloroplast race from the rest of Europe. Subsequently, Voshell et al. [19] were the first to use ITS with plastid *trnT-F* in *Phalaris* to infer a phylogenetic tree for the genus as well as determine floret evolution and polyploidy relationships*.* The shortest ITS region length was 588 bases in *P. rotgesii* to the longest of 602 bases in *P. arundinacea* (both of which are closely related) and 142 of 169 variable characters were parsimony-informative [19]. ITS sequencing differentiated two main clades in the genus with an additional subclade, proving the utility and robustness of ITS in taxonomically distinguishing among species and within species’ genotypic differences as well as discerning ploidy and floret type variation. Subsequent studies using ITS in *Phalaris* [15, 20] were used to determine historic dispersal routes from the center of origin in the Mediterranean Basin into the Americas via the Bering land route. *Phalaris arundinacea* appeared in two ITS clades with one embedded in Europe while the other was distinctly North American; the mid-Miocene was identified as the epoch in which *Phalaris* species diversification occurred [20].

The recent discovery that, based on SNPs, all historic herbaria and extant riparian and cultivated populations of *P. arundinacea* in the State of Minnesota are most likely North American natives [29, 36] provided impetus for the present study to examine specific Minnesota / North American native *P. arundinacea* genotypes for their identity with reported ITS for the species as well as for comparison with additional *Phalaris*. The objectives of this study were to: 1) amplify and compare ITS regions (ITS1 and ITS2) of historic herbaria and extant (fresh) *Phalaris* specimens; 2) analyze ITS regions for species-specific polymorphisms (diagnostic SNPs) and 3) compare ITS regions of historic *Phalaris* specimens with known extant *Phalaris* species. Associated null hypotheses tested, respectively, were: 1) H_o_ = There is no differences in ITS region between herbaria and fresh *Phalaris* specimens due to herbarium sample age; 2) H_o_ = There is no ITS region polymorphism found within *Phalaris* species and 3) H_o_ = There is no additional polymorphism between herbaria genotypes and currently known extant *Phalaris* species in GenBank, the National Center for Biotechnology Information (NCBI) [42].

## Results

### Historic specimen DNA degradation

**Fig. 1:**
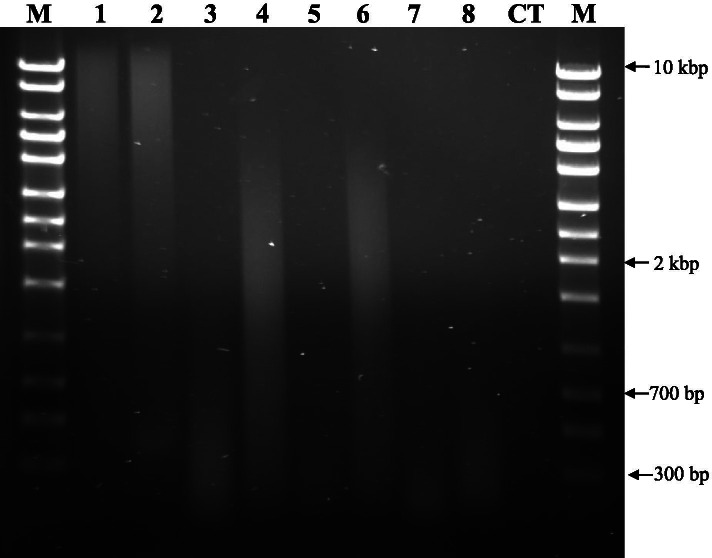
Gel electrophoresis of genomic DNA (200 ng DNA/lane) providing a visualization of DNA degradation. Fresh tissue produced high molecular weight DNA with herbarium specimens producing variable range of DNA degradation, from samples that had DNA highly degraded (< 0.7 kb; 3, 5, 7, 8) to less degraded (fragments in 2–8 kb; 4, 6). Genomic DNA from fresh tissue: (1) *P. aquatica* (PI 476288) and (2). *P. arundinacea* (PI 241065), both > 10 kb*;* from herbarium: (3) *P. canarensis* (619107) and (4) *P. brachystachys* (ISC-V-0021035); herbarium specimens that were PCR re-amplified (5) *P. paradoxa* (ISC-V-0021361) and (6) *P. coerulescens* (ISC-V-0021204). Herbarium specimens with unsuccessful PCR amplifications (7) *P. canarensis* (71229) and (8) *P. minor* (229774). ITS region amplification (fresh and herbarium samples) and re-amplification is presented on Fig. 3. M – DNA size marker; CT – no sample loaded. Full-length blots/gels are presented in Supplementary Fig. 1

Sampling of historic specimens showed variable levels of DNA degradation when compared to DNA obtained from extant *Phalaris* tissue (Fig. 1; Supplementary Fig. 1), similar to previous sampling [29]. Extant tissue DNA extracted from *P. aquatica* (PI 476288) and *P. arundinacea* (PI 241065) (lanes 1 and 2, respectively, Fig. 1) have a majority of higher molecular weight DNA fragments (< 10 kb). Among representative historic samples *P. canarensis* (PI 619107; lane 3, Fig. 1), *P. paradoxa* (ISC-V-0021361; lane 5, Fig. 1), *P. canarensis* (PI 71229; lane 7, Fig. 1) and *P. minor* (PI 229774; lane 8, Fig. 1), all have highly degraded DNA fragments (< 0.7 kb) when compared with other historic specimens, *P. brachystachys* (ISC-V-0021035; lane 4, Fig. 1) and *P. coerulescens* (ISC-V-0021204; lane 6, Fig. 1) with less degraded DNA (2–8 kb).

A standard DNA purification kit (OPS Diagnostics Laboratory, Lebanon, NJ) provided high quality and quantity of genomic DNA that allowed amplification of all extant tissue and most of the historic specimens (41/52 or 78.8%; Table 1). For the majority of herbaria samples, the Optical Density (OD)_260/280_ values were within the expected range for high quality DNA (2.10 ± 0.79, mean ± standard deviation; Table 1) and the second measure of DNA purity (OD _260/230_) had higher variation (1.64 ± 1.41; Table 1). Fresh specimens yielded high quality DNA, based on both the OD_260/280_ (1.93 ± 0.05) and OD _260/230_ (1.93 ± 0.24; Table 2) markers. Both herbaria and fresh specimens have a wider range in OD_260/230_ purity measures. However, for both purity measurements, herbaria specimens were less consistent than fresh specimens.

**Table 1:** *Phalaris* species historic herbaria specimen data from leaf sample collections at the Bell Museum Herbarium (MIN; University of Minnesota) and Ada Hayden Herbarium (ISC; Iowa State University), DNA concentration (ng/μl) of extracted samples, their corresponding DNA quality readings from the spectrophotometer (optical densities [OD] at two ratios: OD _260/280_ and OD _260/230_), amplification success (+/−), herbaria accession codes and the National Center for Biotechnology Information (NCBI) GenBank accession numbers sequences

***Phalaris*** Species	Collection Date	DNA Conc. (ng/μl)	OD_**20/280**_	OD_**260/230**_	Amplification Success	Herbaria Accession Codes	GenBank Accession Number
*P. angusta* Nees ex Trin*..*	16 May 1936	15.8	1.94	1.44	+	ISC-V-0020926	MN811167.1
	16 April 1960	13.8	2.00	1.50	+	ISC-V-0020921	MN811165.1
	3 June 1964	14.5	1.97	1.25	+	ISC-V-0020922	MN811168.1
	12 April 1970	29.2	1.98	1.89	+	ISC-V-0020920	MN811166.1
	3 April 1982	6.1	2.48	1.27	+	ISC-V-0020919	MN811169.1
*P. aquatica* L.	8 September 1935	34.6	1.95	1.79	+	ISC-V-0021399	MN811171.1
	31 August 1959	9.1	2.05	1.13	+	ISC-V-0020927	MN811170.1
	8 July 1962	13.9	1.88	0.81	+	ISC-V-0021398	MN811172.1
*P. arundinacea* L.	30 June 1913	70.8	2.00	1.82	+	71,166	n/a
	28 May 1955	6.9	1.5	0.54	+	532,148	MN811175.1
	27 August 1983	40.7	1.93	2.42	+	753,216	MN811176.1
	26 June 2001	6.3	2.07	2.59	+	484,712	MN811200.1
*P. brachystachys* Link	8 April 1959	10.2	2.12	2.43	+	ISC-V-0021036	MN811180.1
	8 April 1959	10.5	2.37	1.61	+	ISC-V-0021039	MN811179.1
	8 April 1959	63.3	1.91	2.41	+	ISC-V-0021035	MN811178.1
	10 August 1962	7.8	2.21	3.90	+	ISC-V-0021037	MN811181.1
*P. californica* Hook. & Arn.	4 May 1903	16.2	2.03	2.11	+	ISC-V-0021040	n/a
	24 May 1907	10.6	1.47	0.66	–	ISC-V-0021044	n/a
	18 June 1908	19.9	1.86	1.04	+	ISC-V-0021043	MN811182.1
	1958	10.1	2.16	2.52	–	ISC-V-0021041	n/a
	ND*	8.3	1.78	1.79	–	ISC-V-0021042	n/a
*P. canariensis* L.	August 1884	26.1	1.91	2.62	+	71,226	n/a
	29 July 1886	41.9	1.87	2.39	–	71,229	n/a
	12 July 1938	73.1	1.95	2.12	+	367,474	MN811185.1
	July 1969	87.7	1.88	2.09	+	619,107	n/a
*P. caroliniana* Walter	30 May 1940	17.8	2.01	2.07	+	ISC-V-0021097	MN811188.1
	28 May 1964	2.8	2.53	1.74	+	ISC-V-0021081	MN811187.1
	6 April 1973	7.8	2.28	2.47	+	ISC-V-0021166	n/a
	3 May 1982	25.9	1.97	2.17	+	ISC-V-0021080	MN811186.1
*P. coerulescens* Desf.	24 November 1958	23.8	2.05	1.61	+	ISC-V-0021199	MN811190.1
	15 December 1958	5.0	2.60	1.40	+	ISC-V-0021198	n/a
	8 April 1959	22.9	2.06	1.93	+	ISC-V-0021204	n/a
	8 April 1959	6.6	2.19	1.45	+	ISC-V-0021203	MN811189.1
*P. lemmonii* Vasey	1888	6.6	1.61	−4.95	–	ISC-V-0021333	n/a
	18 April 1946	26.2	2.02	1.89	+	ISC-V-0021334	n/a
	15 June 1955	42.9	1.79	0.79	+	ISC-V-0021329	MN811192.1
	7 May 1956	9.0	2.11	0.96	+	ISC-V-0021336	MN811191.1
	23 May 1980	8.8	1.80	0.63	+	ISC-V-0021328	n/a
*P. minor* Retz.	May 1882	12.6	1.92	0.88	–	ISC-V-0021344	n/a
	April 1901	36.3	1.81	2.07	–	229,774	n/a
	12 April 1918	17.0	1.80	0.72	–	ISC-V-0021338	n/a
	24 November 1958	12.7	2.11	1.74	–	ISC-V-0021342	n/a
	8 April 1959	15.3	2.12	1.70	+	ISC-V-0021341	MN811193.1
*P. paradoxa* L.	13 May 1914	19.6	2.03	2.20	+	ISC-V-0021360	n/a
	4 June 1915	10.9	1.71	0.61	–	ISC-V-0021363	n/a
	2 June 1934	71.8	1.68	0.75	+	ISC-V-0021361	MN811195.1
	3 May 1940	3.8	1.90	7.05	–	ISC-V-0021362	n/a
	8 April 1959	2.5	7.42	0.27	+	ISC-V-0021415	MN811194.1
*P. truncata* Guss. ex Bertol.	11 November 1958	19.7	1.88	2.68	+	ISC-V-0021377	MN811198.1
	24 November 1958	7.7	2.25	2.03	+	ISC-V-0021384	MN811199.1
	15 December 1958	30.5	2.06	1.81	+	ISC-V-0021395	MN811197.1
	24 December 1958	2.9	2.66	0.38	+	ISC-V-0021373	MN811196.1

ND* denotes no reported date for herbarium specimen collection

n/a denotes specimens with no GenBanksequence publication

+ denotes a successful PCR amplification (see Materials and Methods section)

- denotes an unsuccessful PCR amplification (see Materials and Methods section)

**Table 2:** Extant (fresh) *Phalaris* species specimens (USDA - GRIN) used for ITS sequencing, DNA concentration (ng/μl) of extracted samples, their corresponding DNA quality readings from the spectrophotometer (optical densities [OD] at two ratios: OD _260/280_ and OD _260/230_), amplification success (+/−), GRIN accession numbers and the published National Center for Biotechnology Information (NCBI) GenBank accession numbers

***Phalaris*** Species	DNA Concentration (ng/ul)	OD _**260/280**_	OD _**260/230**_	Amplification Success	GRIN Accession Number	GenBank Accession Number
*P. aquatica*	60.7	1.97	2.14	+	PI 476287	MN811177.1
	80.7	1.97	2.06	+	PI 476288	n/a
	59.9	1.96	1.93	+	PI 303825	MN811173.1
*P. arundinacea*	52.2	1.95	2.13	+	PI 241065	n/a
	28.5	1.84	1.47	+	PI 422030 (*‘Ioreed’*)	MN811174.1
*P. canariensis*	53.6	1.89	1.99	+	PI 578800	MN811183.1
	57.8	1.97	1.79	+	PI 578798	MN811184.1

n/a denotes specimens with no GenBank sequence publication

+ denotes a successful PCR amplification

- denotes an unsuccessful PCR amplification

**Fig. 2:**
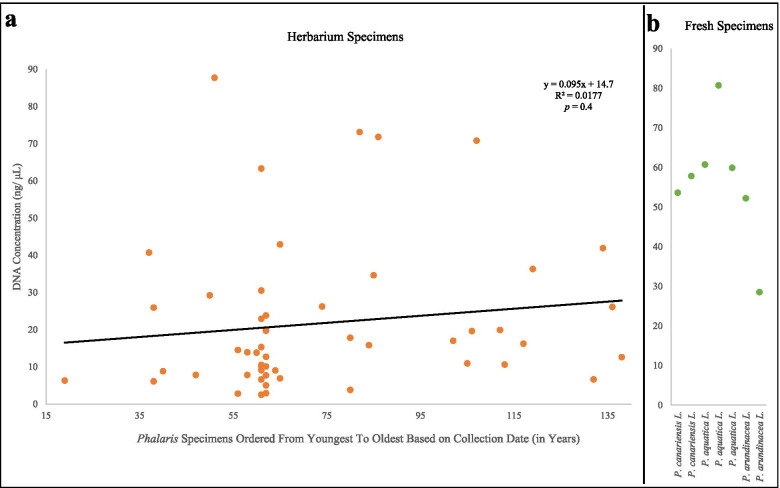
**a** Scatter plot and linear regression of the relationship between DNA concentration (ng/μL) and sample age among herbarium specimens (orange) of *Phalaris* species ordered by relative age (collection year from 1882 to 2001). **b** Extant (fresh) samples harvested on June 17th, 2019; linear regression was not performed due to small sample size

Concentration of DNA from historic *Phalaris* specimens varied widely, from 2.5 ng/μl (*P. paradoxa*, ISC-V-0021415; Table 1) to 87.7 ng/μl (*P. canariensis*, 619,107; Table 1), despite similar amounts of tissue used for extraction. No correlation between the age of the historic samples over a 119 year range and DNA concentration was found, based on the insignificant slope of the linear regression, y = 0.095x + 14.7 and a small but insignificant r^2^ = 0.0177 (*p*-value = 0.4; Fig. 2a). Extant specimens exhibited much more consistent values with a range of 28.5 to 80.7 ng/μl (Table 2). No regression was fit to the concentration of fresh specimens (Fig. 2b) due to an inadequate sample size (*n* = 7). One of the oldest samples collected 29 July 1886 (*P. canariensis*; 71,229, lane 7, Fig. 1; Table 1) produced 41.9 ng/μl of DNA of relatively good quality. This specimen did not, however, yield a successful amplification or sequence of the ITS regions.

### ITS amplification and sequencing of *Phalaris* specimens

**Fig. 3:**
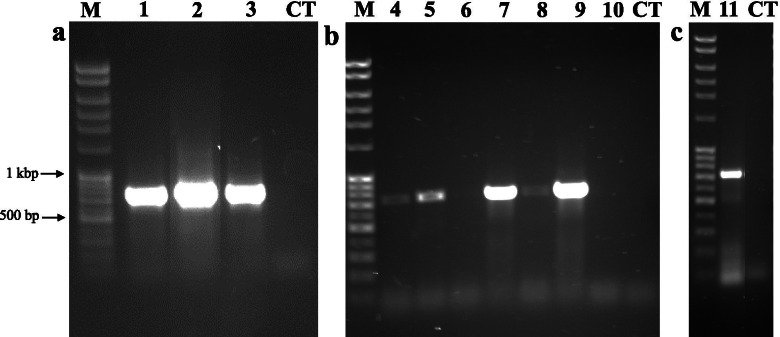
**a** Examples of PCR amplification results of the plant specific ITS specific region [43] on genomic DNA from panel **a**. fresh *Phalaris canarensis* (1; 53.6 ng; PI 578800), *P. aquatica* (2; 60.7 ng; PI 476287) and *P. arundinacea* (3; 28.5 ng; PI 422030); **b** herbarium specimens with a uniform quantity of 50 ng *P. canarensis* (4; 71,226)*, P. californica* (5; ISC-V-0021043), *P. californica* (6; ISC-V-0021040)*, P. caroliniana* (7; ISC-V-0021097), *P. paradoxa* (8; ISC-V-0021360), *P. coerulescens* (9; ISC-V-0021199), *P. minor* (10; ISC-V-0021338); **c** PCR re-amplification result of purified PCR reaction of *P. canarensis,* 1/50 of previous PCR reaction (B4) as a PCR template*.* M – DNA ladder, CT – Control. Full-length blots/gels are presented in Supplementary Fig. 2

Plant-specific ITS-P5 and ITS-U4 primers were efficient in PCR amplification of the ITS regions across a diverse set of *Phalaris* species, including a majority of *Phalaris* tissue extracted from herbarium specimens, producing complete sequences of both the ITS1 and ITS2 regions (31/52 or 59.6%, Table 1). The oldest specimen fully sequenced was collected in 1908: *P. californica* (ISC-V-0021043, MN811182.1; Table 1). In addition, most of the ITS amplifications were suitable for direct sequencing after clean up. Overall, 31/41 (75.6%) herbarium (+; Table 1) and 5/7 (71.4%) fresh specimens (+; Table 2) produced full sequences of the ITS region for 12 *Phalaris* species. An additional six sequences of fresh (*n* = 2; *P. arundinacea* PI 241065 and *P. aquatica* PI 476288) and herbaria samples (*n* = 4; *P. arundinacea* 71,166*, P. canariensis* 619,107 and 71,226 and *P. caroliniana* ISC-V-0021166) produced partial sequences of the ITS region but were not used for sequence analysis. High PCR amplification success for fresh specimens were evidenced across the sampling, i.e. *P. canariensis* (lane 1; Fig. 3a; Supplementary Fig. 2), *P. aquatica* (lane 2; Fig.3a; Supplementary Fig. 2) and *P. arundinacea* (lane 3; Fig. 3a; Supplementary Fig. 2).

The low PCR amplification success rate of herbaria specimens is most likely caused by DNA degradation that did not allow amplification of the ITS DNA, as found for samples *P. californica* (ISC-V-0021040), *P. paradoxa* (ISC-V-0021360) and *P. minor* (ISC-V-0021338; lanes 6, 8,10, respectively; Fig. 3b; Supplementary Fig. 2). Overall, herbaria specimens that were collected before 1915 ± 28 (mean ± S.D.) years did not amplify the full ITS region, wherein most of the *Phalaris* herbaria samples collected later, in 1953 ± 23 years, were successful in ITS region amplification (Table 1). The 50 ng/μl DNA template was used as a standard DNA concentration for PCR amplification of all *Phalaris* herbarium specimens (Fig. 3b). However, due to variable degradation of DNA from herbarium specimens, ITS amplification did not always allow use of those products for direct sequencing based on sequencing requirements (Fig. 3b, lane 8; University of Minnesota Genomics Center – Sanger Sequencing Classic). For some herbaria samples it was necessary to reamplify PCR products, which allowed for later product sequencing (Fig. 3c; Supplementary Fig. 2).

### Polymorphism analysis of *Phalaris* species

**Fig. 4:**
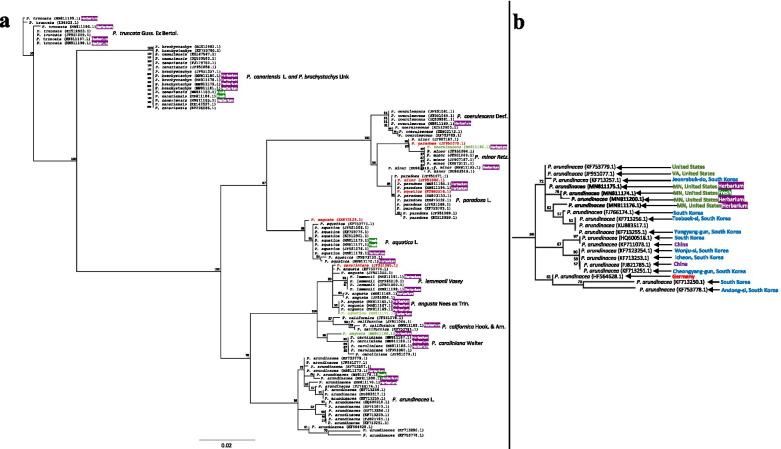
Distance tree representing distance between GenBank sequences of complete ITS region for the twelve *Phalaris* species included in this study and sequences generated from herbaria and fresh samples in this investigation through sequence alignment (Supplementary Fig. 3); **a** displays the whole tree with GenBank sequences that do not follow proper species branching assignment in red text and green text signifies sequences generated in this investigation from herbarium specimens that do not follow proper species branch assignment. **b** The *P. arundinacea* clade is expanded with specimen geographic collection location indicated from available location information on GenBank

Reconstruction of genetic distance from newly sequenced herbaria and fresh *Phalaris* specimens with combination of those available in GenBank showed proper specimen classification and grouping within the same species (Fig. 4a; Supplementary Fig. 3). Generally, the sequences produced in this study matched with previous sequences of the same species. Shared SNPs from the matching sequences created unique clades by species in the distance tree (Fig. 4a). Some species (*P. brachystachys* and *P. canariensis*) are more closely related than others and the distance between clades suggests that the ITS region was insufficient to separate those two species into separate branches. Both species share nine unique SNPs that distinguish the two from the rest of *Phalaris* (Supplementary Fig. 3; Table 3).

**Table 3:** Diagnostic, species-specific, single nucleotide polymorphism (SNP) sites in the ITS1 and ITS2 regions (with the species-specific letter code followed by the regions numbered 1–9) for *Phalaris* species, within each species-specific site, specifying the nucleotide substitution (A/C, A/G, A/T, C/A, C/G, C/T, G/A, G/C, T/A, T/C, T/G) and the relative position each site on the ITS1 and ITS2 multialignments (Supplementary Fig. 4). Two species (*P. lemmonii, P. angusta*) lack diagnostic SNPs, while two species (*P. brachystachys, P. canariensis*) are indistinguishable (N/A)

***P. truncata*** **Guss. ex Bertol.**	**T1** A/C _23_	**T2** G/C _155_	**T3** T/G _166_	**T4** T/A or T/C _245_	**T5** T/A or T/G _381_				
***P. brachystachys*** **Link &** ***P. canariensis*** **L.**	**B-C1** A/G 52	**B-C2** C/T 65	**B-C3** A/C 88	**B-C4** A/C 162	**B-C5** C/A 246	**B-C6** T/C _315_	**B-C7** A/C _339_	**B-C8** A/G _346_	**B-C9** A/G _349_
***P. aquatica*** **L.**	**A1** T/G 45	**A2** A/T 132	**A3** A/T or A/C 221	**A4** C/T or C/A 237	**A5** A/C 352				
***P. coerulescens*** **Desf**	**C1** DEL 426	**C2** T/C 424	**C3** A/T 414						
***P. minor*** **Retz.**	**M1** T/C 202	**M2** T/G or T/A 252	**M3** T/C 373						
***P. paradoxa*** **L**	**P1** C/G 403								
***P. californica*** **Hook. & Arn.**	**CA1** G/A or G/C 246	**CA2** G/A 416							
***P. caroliniana*** **Walter**	**CR1** A/T 213	**CR2** T/A 385	**CR3** A/G 381						
***P. arundinacea*** **L.**	**AR1** T/G or T/C 198	**AR2** C/T 215	**AR3** A/G or A/T 252	**AR4** G/A 281	**AR5** A/C 351	**AR6** T/C 337			
***P. lemmonii*** **Vasey**	N/A								
***P. angusta*** **Nees ex Trin.**	N/A								

A large *Phalaris* species sequence collection allowed us to identify species-specific SNPs that identified selected *Phalaris* species, based on single SNPs found within ITS1 and ITS2 (Table 3). Two additional species (*P. lemmonii* and *P. angusta*) lacked species-specific SNPs, based on their sequence alignment (Table 3).

Considerable polymorphism was exhibited in the *P. arundinacea* clade (Table 3; AR1, AR2, AR3, AR4, AR5 and AR6 diagnostic, species-specific, SNP sites in the ITS1 and ITS2 regions), particularly among Asian and those of N. American origin (Fig. 4b). We did not observe sequence abnormalities between herbaria specimens and fresh tissue specimens of the same species despite suspected misidentified samples. Collection locations of *P. arundinacea* specimens reflect the relatedness within or distinctiveness among *P. arundinacea* genotypes (Fig. 4b). The *P. arundinacea* ITS sequences identified three clusters, the first with *P. arundinacea* originating in the United States which are N. American in origin [29, 36]: MN81175.1, MN811200.1, MN811176.1 (herbarium) and MN811174.1 (fresh) clustered tightly together (Fig. 4b), although they were grouped in between Korean accessions KF713257.1 and FJ766174.1. The second cluster of *P. arundinacea* was an adjacent clade, on one side of the N. American natives, which included one genotype from Korea (KF713257.1, Fig. 4b) and two others from the United States and the east coast USA State of Virginia (KF753779.1, JF951077.1). On the other side in closely related clades were four Eurasian genotypes from Taebaek-si and Yungyang-gun, S. Korea, specifically FJ766174.1, KF713256.1, KU883517.1 and KF13255.1 (Fig. 4b). A third, more distant cluster of two inter-related clades contained strictly Eurasian types from S. Korea (*n* = 5), China (*n* = 2) and Germany (*n* = 1) (Fig. 4b). The relatedness, based predominantly on geographic distribution, illustrates high variation of the *P. arundinacea* samples within the ITS region along with the possibility to distinguish among *P. arundinacea* from different geographic locations on a continental scale (Fig. 4b).

The composition of the *Phalaris* distance tree also indicates that there are GenBank sequences and herbaria specimens that may have been misidentified (Fig. 4a). In addition to proper classification and groupings, multiple ITS sequences found in GenBank did not follow the species branch assignment: *P. angusta* (KX873129.1) was classified with the *P. aquatica* clade; *P. paradoxa* (JF951070.1) was assigned to *P. minor* clade; *P. caroliniana* (JF951065.1) was assigned to *P. angusta* clade; *P. minor* (JF951086.1) and *P. aquatica* (KU883516.1) assigned to the *P. paradoxa* clade (Fig. 4a). Three of the sequences produced in this research did not group with a specific clade: *P. coerulescens* (MN811190.1; ISC-V-0021199), *P. aquatica* (MN811171.1; ISC-V-0021399) and *P. angusta* (MN811166.1; ISC-V-0020920), which clustered with *P. minor*, *P. angusta* and *P. caroliniana*, respectively. Both MN811189.1 and MN811190.1 (that did not group with a species clade) were *P. coerulescens* herbarium specimens grown from Turkish seed at Iowa State University but did not have the same SNPs, further suggesting possible misidentification. The misidentification is likely due to the similar morphological characteristics of the *Phalaris* genus and highlights the need for ITS barcoding to distinguish among and between extant and historic *Phalaris* species. It also highlights the need to exercise stringent quality control measures on newly generated sequence data and to approach public sequence databases in a critical way.

## Discussion

### Historic specimen DNA degradation

Efficient DNA amplification (barcoding) requires the DNA template to be of high quality (measured by OD_260/280_ and OD_260/230_), quantity (ng of DNA template/μl) and integrity (visualized on agarose gels). High DNA quality equates to the DNA being free from protein and chemical contaminants with a sufficient quantity of DNA template to start PCR amplification of targeted DNA regions [44]. A measure of DNA quality is the ability to be amplified. A second round of PCR can reduce inhibitors, as sometimes found in extracted DNA [45]. The potential effect of PCR inhibitors is reduced during the PCR reaction, in that isolated DNA from each reaction was diluted 1/25 in volume prepared for PCR in our experimentation. One of the most critical aspects when working with herbaria specimens is physical DNA degradation (fragmentation) that directly impacts amplification of selected DNA regions, such as the ITS regions.

In the case of historic *Phalaris* specimens [29], the additional factor of varying levels of genomic DNA degradation also affect DNA amplification [45]. Choi [46] demonstrated that trends between age and purity were not generally significant in four evolutionarily, geographically and ecologically different plant lineages. Therefore, DNA purity of specimens is likely due to the post-harvest and storage techniques used. Historic samples that are preserved and stored in traditional herbaria cases maintain the plant tissue appearance and overall specimen condition to minimize tissue degradation and maintain the phenotype of the type specimens as illustrated in Supplementary Fig. 4 [38]. However, the process and conditions of tissue preservation (harvest and post-harvest tissue storage) may not preserve DNA integrity [29, 47], leading to degradation of DNA in herbarium specimens which reduces amplification of ITS regions for samples with significantly degraded DNA [48]. Other factors, such as sample age, are also inadequate assessors of DNA quality [49]. Nonetheless, despite obtaining ODs with high purity and DNA concentrations of 2.5 to 87.7 ng/μl (Table 1), only some of the *Phalaris* herbaria samples were extracted with high molecular weight DNA (Fig. 1) which was not as consistent as with fresh (extant) specimens, yielding 28.5 to 80.7 ng/μl (Table 2).

It is not sufficient to obtain a certain quantity of DNA template (i.e. 50 ng/PCR reaction), but evaluation of DNA degradation should also be taken into account as a major factor limiting proper PCR amplification [43, 50]. Another tactic to be considered is to amplify the genetic marker in a stepwise fashion when the DNA template is highly degraded [48]. Additionally, DNA reconstruction is a method that could be employed early on to increase DNA quality (OD _260/280_ ratios) and later PCR success and sequence length [51]. While an exhaustive study was not performed on reamplification options (nested PCR, whole genome amplification, or others) with the purpose of increasing the concentration of the PCR product to the required concentration for Sanger sequencing, it was found that a visible band was needed for sequencing. This method could be useful when working with old or precious tissue, such as herbarium, as a means to increase sequencing success.

The lack of consistency in herbaria specimens may be due to increased degradation and/or minor levels of contaminants, although the wide age range of 119 years across herbaria samples was not correlated with DNA concentration (Fig. 2a). When using the ITS region for barcoding herbaria specimens within the Juncaceae, a significantly negative association between age and sequencing success was found [47]. Therefore, the age relation to sequencing success may vary by species as well as storage treatments for herbarium specimens, rather than age – at least in the case of our *Phalaris* herbaria samples.

### ITS amplification and sequencing of *Phalaris* specimens

Complete ITS1 and ITS2 sequences were obtained in 59.6% of the accessions across 12 species in the genus *Phalaris* (Tables 1, 2) and were within the expected range of 757 ± 140 bases [52]. The oldest herbarium specimen that was fully sequenced was *P. californica* collected in 1908 (Table 1). Six fresh and herbarium partial sequences of *P. arundinacea, P. aquatica, P. canariensis* and *P. caroliniana* (Tables 1, 2) were not used in the sequence analyses. The occurrence of partial sequences is not unusual, as previous researchers had difficulties with *P. peruviana* producing low quality sequences with missing data [15]. Design of a new set of ITS PCR primers that would be located farther from the beginning (and end) of the ITS region would most likely provide more high quality ITS sequences. Sanger sequencing tends to produce low quality sequences, just after the end of the sequencing primer position [53], that should be removed during sequence quality review.

Other factors can contribute to sequencing success such as age, preservation procedure, specimen color, the climate collected in and taxon related plant characteristics [53–55]. While Brewer et al. did not investigate the Poaceae family, they did determine that sequencing success can be related to taxon-specific traits in relation to the preservation technique used (herbaria vs. silica gel-dried specimens) [54]. The Poaceae family that the *Phalaris* genus belongs to was found to have a decreased sequence recovery of the ITS2 region with age and silica gel vs. herbaria preservation method [55]. This calls for the optimization of taxon-specific traits for species-focused studies such as this to achieve maximized sequence retrieval for barcoding.

### Polymorphism analysis of *Phalaris* species

Multiple sequence alignment of the ITS region from historic herbaria and fresh *Phalaris* species showed that sequences and DNA polymorphisms found in newly sequenced *Phalaris* species resemble those previously reported [19, 56]. The similarity among the phylogenetic tree of the ITS region constructed by Voshell et al. [19] (fresh *Phalaris* tissue) and the distance tree constructed in this study (mainly herbaria *Phalaris* tissue) verifies the relatedness found among the *Phalaris* species and the genetic distinctions that can be extrapolated from SNPs in the ITS region with the use of herbarium tissue as old as 119 years.

Two species (*P. lemmonii, P. angusta*) lacked species-specific SNPs based on their sequence alignment (Table 3). Their relatedness may be expressed in flower morphology and may be due to geographic separation [19, 56]. In other instances, some *Phalaris* species, like *P. brachystachys* and *P. canariensis,* were too closely related such that the ITS region was insufficient to separate them (Fig. 4a). To separate both species we recommend using other barcoding regions, such as atpF–atpH, matK, rbcL, rpoB, rpoC1, psbK–psbI and trnH–psbA to see if a SNP can be found [57].

When comparing the phylogenetic tree constructed by Voshell et al. [19] to our distance tree (Fig. 4) of predominantly herbarium based ITS regions, similar clade groupings can be identified. A separation of clades (*P. canariensis, P. brachystachys* and *P. truncata*) and remaining species was identified in our study (Fig. 4), similar to [19]. The trees from Fig. 4, Supplementary Fig. 3 and Voshell et al. [19] form a consistent cluster of the species *P. lemmonii, P. angusta, P. caroliniana, P. californica* and *P. arundinacea* interpreted to resemble lineages 1 and 2 of the *Phalaris* genus [19]. The divergence of lineage 3 including *P. coerulescens, P. minor, P. paradoxa* and *P. aquatica* was also found in this analysis [19]. The similarity between the ITS inferred distance in this study (mainly herbaria *Phalaris* tissue) and by Voshell et al. [19] (fresh *Phalaris* tissue) verifies the relatedness found among *Phalaris* species and the genetic distinctions that can be extrapolated from SNPs in the ITS region with the use of herbarium tissue as old as 112 years (MN811182.1). No false SNPs were produced in sequence alignment (Supplementary Fig. 3) and distance tree construction due to the age of tissue and relatedness [19], indicating the versatility of herbarium specimens in ITS barcoding (Fig. 4a). Nucleotide polymorphism exists among most *Phalaris* species that were shared among historic and extant tissue types.

The clustering of *P. arundinacea* ITS sequences into predominantly regional clades from the USA and Asia is a notable grouping (Fig. 4b). All genotypes from N. America were in a single clade, distinct from most other ones of Eurasian origin. Despite considerable polymorphisms in the species (AR1-AR6, Table 3), a tight and adjacent clustering of the four native N. American *P. arundinacea* accessions from Minnesota (extant and historic MN811175.1, MN811200.1, MN811176.1, MN811174.1; Fig. 4b) was unexpected. These were grouped among two accessions of Korean origin (KF713257.1 and FJ766174.1; Fig. 4b) which could be the original ancestors of *P. arundinacea* germplasm that came across the land bridge of the Bering Strait into the State of Alaska, USA [20] during the late Tertiary period [21]. Other genotypes of USA origin (either native N. American or of Eurasian ancestry) were separated in the clade from the four N. American (Minnesota) natives. While the separation of USA and Asia *P. arundinacea* was not 100% consistent, most of the Eurasian types were in distinct clades; the use of additional barcoding markers would most likely allow proper geographic association of *P. arundinacea.* The relatedness of N. American vs. most Eurasian types illustrates the possibility to distinguish among *P. arundinacea* from different geographic locations on a continental scale (Fig. 4b). Future research is essential with our large germplasm base of native riparian, roadside, lakebed and cultivated N. American types in the Midwest (> 3000 genotypes) [29, 36] to potentially provide substantive confirmation of this distinction or a melding of all N. American types.

The high variation within the *P. arundinacea* and the detection of variants, forma and subspecies (Fig. 4b) further justifies the sequencing of more specimens within the ITS region and other barcoding regions [56–60] to better understand the relatedness and variation within this species and the genus as a whole. Herbaria curators at The Consortium of California Herbarium have recognized the *P. arundinacea* species to contain the subspecies *picta* and *typica*, four varietas (*arundinacea, colorata, geuina,* and *japonica*) and nine forma (*Arundinacea, coarcta, luteo-Picts, minor, pallens, pallida, Ramifera, Ramosa, variegata;* http://www.cch2.org/portal/taxa/index.php?taxon=Phalaris+arundinacea&formsubmit=Search+Terms). Further genetic structure analysis of *P. arundinacea* will provide the potential ability to genetically distinguish *P. arundinacea* by continents, even though it is morphologically indistinguishable. More exhaustive analysis of the ITS region may provide SNPs to discriminate the native vs. exotic status in N. American populations. Furthermore, the secondary structure of the ITS1 and ITS2 regions may provide a more accurate and detailed idea of the relatedness, sequence evolution and barcodes of the Phalaris species – especially when used in combination with sequence data [61].

## Conclusions

Our study demonstrates that most *Phalaris* herbaria specimens can be used to produce high quality ITS sequences that are useful to evaluate past genetic resources. The methods used in this study are relatively simple to replicate and, most importantly, do not require specific protocol modifications. Overall, the use of a standard DNA isolation protocol, PCR amplification method and direct PCR product sequencing were sufficient to sequence the majority of the collected herbarium specimens. The lack of amplification of some specimens is most likely due to high DNA degradation. Furthermore, herbaria can provide the plant tissue and taxonomically identified specimens, facilitating the identification of new species-specific barcodes and evaluation of past genetic resources. The ITS region (ITS1 and ITS2) was sufficient to distinguish eight out of twelve *Phalari*s species in this study. In addition, separation of the genus and within species was possible, implying that subsampling of multiple genotypes of this same species is necessary. The ITS region distinguished among most Eurasian vs. N. American accessions of *P. arundinacea.* A further development of within species and genus specific barcodes could contribute to designing PCR primers for efficient and accurate plant identification. Our finding of misidentified *Phalaris* species indicates the need for the DNA sequence database curation for proper specimen identification.

## Methods

### Germplasm collection

Extant (fresh) and historic herbaria samples of the *Phalaris* species were used for genomic DNA extraction. A total of *n* = 52 historic specimens were collected from the Bell Museum Herbarium, University of Minnesota, St. Paul, MN (MIN; *n* = 9; Table 1) and the Ada Hayden Herbarium, Iowa State University, Ames, IA (ISC; *n* = 43; Table 1). Formal identification of each voucher specimen had already been accomplished by each herbaria curator. Destructive sampling of ~ 2.5 × 0.63 cm of leaf tissue was performed on each herbarium specimen for leaf samples positioned in the back of the specimen to not decrease their visual integrity, similar to our previous methodology [29]. Permission for destructive tissue sampling was obtained from each herbarium curator in advance. Specimen notes were added to delineate the specimen-specific sampling. This herbarium collection represents a range of North American *Phalaris* herbarium specimens with collection dates ranging from 1882 (*P. minor* Retz*.*; ISC-V-0021344) to 2001 (*P. arundinacea* L*.*; 484,712; Table 1). Selection of the *P. arundinacea* herbaria samples were already tested for SNP genetic variation and provided sufficient and high quality nuclear DNA could be extracted, were determined to be most likely native North American genotypes [29, 36].

For additional sampling purposes, seeds were obtained from the U.S. Department of Agriculture Germplasm Resources Information Network (USDA GRIN; https://www.ars-grin.gov/). Formal identification of each seed specimen had already been accomplished by the USDA GRIN taxonomist; no voucher specimens were deposited since the seed is part of the national USDA GRIN system. Germinated seedlings were used as extant specimens of the *Phalaris* species. Fresh samples served as an amplification and SNP detection validation. Extant specimens included three *P. aquatica* (PI 476287; PI 476288; PI 303825), two *P. arundinacea* (PI 241065; PI 422030) and two *P. canariensis* (PI 578800; PI 578798; Table 2). Seeds were sown in 10 cm square pots filled with Sungro Professional Growing Mix (Sun Gro Horticulture; SKU:5105, Agawam, MA) and placed in a mist house (greenhouse with an intermittent mist system). Once the seedlings germinated and true leaves were developed, plants were moved to the greenhouse for continued growth and leaf harvest. Environmental conditions in both greenhouses were 24.4 ± 3.0/18.3 ± 1.5 °C day/night daily integral and a 16 h long day photoperiod (0600–2200 HR) lighting (400 W high pressure sodium high intensity discharge lamps, HPS-HID) at a minimum of 150 μmol m^− 2^ s^− 1^. Plants were fertilized twice daily, between 0700 and 0800 and 1600–1700, using a constant liquid feed (CLF) of 125 ppm N from water-soluble 20 N–4.4P–16.6 K (Scotts, Marysville, OH). Fungicide drenches were applied in monthly rotations. Leaf tissue of matured, fully-expanded leaf trips were harvested and stored at − 20 °C in sealed plastic bags.

### GenBank ITS resources

National Center for Biotechnology Information (NCBI) GenBank database searches were performed to collect existing ITS region sequences for comparative purposes from the twelve *Phalaris* species included in this study (Table 1). In this research, only *Phalaris* ITS sequences that contained full ITS1 and ITS2 regions were included and partial sequences were not compared. Partial sequences were not included because missing sequences could contain SNP polymorphism(s) that would contribute to less accurate distance analysis. Multiple ITS sequences from twelve *Phalaris* species were found (*n* = 68, full sequences): *P. angusta* (KX873129.1, KF753774.1, JF951055.1, JF51054.1), *P. aquatica* (KU883516.1, KF753775.1, JF951056.1, KF753776.1, KC512901.1, JF951076.1, KX873130.1), *P. arundinacea* (KF753779.1, JF951077.1, KF713257.1, FJ766174.1, KF713256.1, KU883517.1, KF713255.1, HQ600518.1, KP711073.1, KF713254.1, KF713253.1, FJ821785.1, KF713251.1, HF564628.1, KF713250.1, KF753778.1), *P. brachystachys* (KC512902.1, KF753780.1, JF951057.1), *P. californica* (JF951078.1, JF951064.1, KF753781.1), *P. canarensis* (KX147547.1, DQ539580.1, FJ178782.1, JF951058.1, KX147537.1, KP296086.1), *P. caroliniana* (JF951065.1, JF951080.1, JF951079.1), *P. coerulescens* (JF951081.1, JF951066.1, DQ539581.1, KC512900.1, HE802172.1, KF753782.1), *P. lemmonii* (MF964010.1, JF951082.1), *P. minor* (JF907187.1, JF951084.1, JF951069.1, JF907187.1, KX873131.1, KU883518.1, JF951086.1), *P. paradoxa* (JF951070.1, JF951071.1 KX873133.1, KX873132.1, JF951088.1, KF753783.1, JF951089.1, KC512899.1) and *P. truncata* (L36522.1, KC512903.1, JF951059.1). The geographic source of the *P. arundinacea* specimens were recorded if information was available on GenBank or in the associated published work.

### DNA extraction

Nuclear DNA was extracted from historic and extant *Phalaris* samples using Synergy 2.0 Plant DNA Extraction Kit (OPS Diagnostics Laboratory, Lebanon, NJ) with minimal adjustments to the protocol [29]. Tissue was loaded using scissors and forceps that were washed in soapy water, rinsed twice in distilled water and dried with paper towels. Extant samples, unlike historic ones, were kept cool on dry ice throughout the loading process. Samples were ground for a total of 15 min at 1500 rpm in homogenizer (Geno/Grinder; SPEX SamplePrep, Metuchen, NJ). Once purified, DNA was suspended in molecular grade water and stored at − 20 °C. DNA quality and quantity were checked with a spectrophotometer (NanoDrop 2000; Thermo Scientific, Waltham, MA). Quality guidelines were followed as recommended by Thermo Fisher Scientific as ~ 1.8 O _260/280_ and 1.8–2.2 O _260/230_ considered as “pure” DNA (NanoDrop 2000/2000c Spectrophotometer V1.0 User Manual, 2009). Genomic DNA was visualized on 1% (w/v) agarose gels (1 × Tris-acetate-EDTA buffer) and stained with ethidium bromide, 6X loading dye (New England BioLabs; Ipswich MA) and a DNA ladder (FullRanger 1 kb; Norgen Biotek Corp, Thorold, ON, Canada). Only herbarium specimens with the highest quantity of DNA were used to visualize degradation (200 ng / lane on an agarose gel) due to the limited DNA.

To calculate correlation between herbarium tissue age and the concentration of genomic DNA obtained and to the PCR amplification success from herbarium tissue Microsoft Excel 365 (Microsoft, Redmond, WA) with Regression function was used to estimate *p*-value.

### PCR amplification

Polymerase chain reaction (PCR) was performed using 10 μM of ITS-P5 and ITS-U4 primers [52]. PCR master mix (GoTaq Green Master Mix, M712; Promega, Madison, WI) plus 1 μL of DNA (with a minimum concentration > 50 ng/μL) or DNA volume was adjusted to obtain 50 ng total DNA. The PCR protocol followed previous methodology [52]: 94 °C for 4 min, then 34 cycles of 30 s at 94 °C, 40 s at 55 °C and 1 min at 72 °C, finishing with 10 min at 72 °C. PCR reactions were visualized using electrophoresis on a 1% (w/v) agarose gel (1 x Tris-acetate-EDTA buffer) with ethidium bromide and a DNA ladder (FullRanger 100 bases; Norgen Biotek Corp, Thorold, ON, Canada). Expected DNA amplification product of ITS-P5 and ITS-U4 primers was 757 ± 140 bases [52]. If a single amplification product was observed, amplified products were directly purified using PureLink™ Quick PCR Purification Kit (Thermo Fisher Scientific, Waltham, MA) and prepared for Sanger sequencing at the University of Minnesota Genomics Center, following UMGC sample requirements (University of Minnesota Genomics Center – Sanger sequencing Classic, http://genomics.umn.edu/sanger-sequencing-classic.php). If the purified PCR reaction yielded less than the total DNA required for Sanger sequencing (25 ng/μL), the PCR product was diluted in water (1/50) and 1 ul of diluted product was used and re-amplified following the same procedure (ITS-P5 and ITS-U4, primer set, PCR reaction composition, PCR program).

### Sequence analysis

Sequencing results were edited and quality-checked using 4Peaks software (Nucleobytes, Alsmeer, Netherlands*;* http://nucleobytes.com/4peaks/) with additional manual sequence trimming. Sequence editing, alignments, annotations and manipulations were done using Geneious 11.1.5 software (Biomatters, Ltd., New Zealand; https://www.geneious.com) [58]. The *Arabidopsis thaliana* (× 52320.1) ITS sequence was initially used to annotate full ITS regions of the newly obtained (*n* = 36) *Phalaris* DNA sequences (Tables 1, 2). Genetic distance among *Phalaris* species was inferred using the Neighbor-Joining method [59] and a bootstrap test was performed for each tree (with 100 replicates) among newly obtained and GenBank ITS sequence collection. A multiple sequence alignment was performed with use of MUSCLE alignment with version 3.8.425 on Geneious software [60]. Diagnostic, species-specific SNPs that differentiate among *Phalaris* species were determined based on multiple alignment of full-length ITS (ITS1 and ITS2 regions) sequences with exclusion of 5.8S ribosomal subunit. Sequences obtained in this study were deposited into the GenBank database. Accession numbers can be found in Tables 1 and 2.

## Supplementary Information

**Additional file 1: Supplementary Figure 1.** Original uncropped image of gel electrophoresis of 200 ng of genomic DNA in Fig. 1. Where lanes 2 and 3 are fresh tissue *P. aquatica* (PI 476288) and *P. arundinacea* (PI 241065) respectively. Remaining lanes (4–9) are from herbarium tissue consisting of *P. canarensis* (619107), *P. brachystachys* (ISC-V-0021035), *P. paradoxa* (ISC-V-0021361), *P. coerulescens* (ISC-V-0021204), *P. canarensis* (71229) and *P. minor* (229774). Lanes 1 and 11 are a DNA size marker (FullRanger DNA ladder 1 kb) while lane 10 is a control lane with no sample loaded. Dotted white line over image illustrate where the image was cropped to form Fig. 1.

**Additional file 2: Supplementary Figure 2.** Original uncropped image of gel electrophoresis of PCR amplification examples of the plant specific ITS region in Fig. 3 [51] where panel labels (a, b, c) correspond. Panel a consist of fresh *P. canarensis* in lane 2 (53.6 ng; PI 578800), *P.* in lane 3 (57.8 ng; PI 578798), *P. aquatica* in lane 4 (60.7 ng; PI 476287), *P. aquatica* in lane 5 (80.7 ng; PI 476288), *P. aquatica* in lane 6 (59.9 ng; PI 303825), *P. arundinacea* in lane 7 (52.2 ng; PI 241065), *P. arundinacea* in lane 8 (28.5 ng/μl; PI 422030), *P. canarensis* in lane 11 (20 ng; PI 578800), *P. canarensis* in lane 12 (20 ng; PI 578798), *P. aquatica* in lane 13 (20 ng; PI 476287), *P. aquatica* in lane 14 (20 ng; PI 476288), *P. aquatica* in lane 15 (20 ng; PI 303825), *P. arundinacea* in lane 16 (20 ng; PI 241065) and *P. arundinacea* in lane 17 (20 ng; PI 422030). Panel b consists of herbarium specimens of uniform 50 ng quantity where lane 21 is *P. canarensis* (71226)*,* lane 22 is *P. californica* (ISC-V-0021043), lane 23 is *P. californica* (ISC-V-0021040)*,* lane 24 is *P. caroliniana* (ISC-V-0021097), lane 25 is *P. paradoxa* (ISC-V-0021360), lane 26 is *P. coerulescens* (ISC-V-0021199) and lane 27 is *P. minor* (ISC-V-0021338). Panel c contains PCR re-amplification results of 1/50 dilutions of purified PCR reactions with *P. canarensis* in lane 31 (484712), *P. californica* in lane 32 (ISC-V-0021043), *P. californica* in lane 33 (ISC-V-0021040), *P. caroliniana* in lane 34 (ISC-V-0021081), *P. truncata* in lane 35 (ISC-V-0021373), *P. truncata* in lane 36 (ISC-V-0021384) and *P. paradoxa* in lane 37 (ISC-V-0021361). Lanes 9, 18, 28 and 38 are control lanes with no sample loaded. Lanes 1, 10, 19, 20, 29, 30 and 39 are a DNA size marker (FullRanger DNA ladder 100 bases). Dotted white line over image illustrate where the image was cropped to form Fig. 3.

**Additional file 3: Supplementary Figure 3.** Sequence alignment used for the distance tree in Fig. 4 with indication of specific single nucleotide polymorphism for *Phalaris* species.

**Additional file 4: Supplementary Figure 4.** Contrast between fresh *P. arundinacea* (PI 422030; MN811174.1) in panel a with a historic herbarium specimen of *P. arundinacea* (753,216; MN811176.1) from the University of Minnesota Herbarium in panel b.

## Data Availability

The DNA sequences produced are deposited in the NCBI GenBank data (https://www.ncbi.nlm.nih.gov/nuccore/). Sequence lists can be found in the Materials and Methods section (Tables 1 and 2) and are listed in the format below of, “***Phalaris Species*****:** herbarium accession code or GRIN accession number, GenBank accession number;”. Specimens without a GenBank accession number are labeled with “n/a”. ***P. angusta*** **Nees ex Trin:** ISC-V-0020926, MN811167.1; ISC-V-0020921, MN8111655.1; ISC-V-0020922, MN811168.1; ISC-V-0020920, MN811166.1; ISC-V-0020919, MN811169.1. ***P. aquatica*** **L.:** ISC-V-0021399, MN811171.1; ISC-V-0020927, MN811170.1; ISC-V-0021398, MN811172.1; PI 476287, MN811177.1; PI 476288, n/a; PI 303825, MN811173.1. ***P. arundinacea*** **L.:** 71166, n/a; 532148, MN811175.1; 753216, MN811176.1; 484712, MN811200.1; PI 241065, n/a; PI 422030, MN811174.1. ***P. brachystachys*** **Link:** ISC-V-0021036, MN811180.1; ISC-V-0021039, MN811179.1; ISC-V-0021035, MN811178.1; ISC-V-0021037, MN811181.1. ***P. californica*** **Hook. & Arn.:** ISC-V-0021040, n/a; ISC-V-0021044, n/a; ISC-V-0021043, MN811182.1; ISC-V-0021041, n/a; ISC-V-0021042, n/a. ***P. canariensis*** **L.:** 71226, n/a; 71229, n/a; 367474, MN811185.1; 619107, n/a; PI 578800, MN811183.1; PI 578798, MN811184.1. ***P. caroliniana*** **Walter:** ISC-V-0021097, MN811188.1; ISC-V-0021081, MN811187.1; ISC-V-0021166, n/a; ISC-V-0021080, MN811186.1. ***P. coerulescens*** **Desf.:** ISC-V-0021199, MN811190.1; ISC-V-0021198, n/a; ISC-V-0021204, n/a; ISC-V-0021203, MN811189.1. ***P. lemmonii*** **Vasey:** ISC-V-0021333, n/a; ISC-V-0021334, n/a; ISC-V-0021329, MN811192.1; ISC-V-0021336, MN811191.1; ISC-V-0021328, n/a. ***P. minor*** **Retz.:** ISC-V-0021344, n/a; 229774, n/a; ISC-V-0021338, n/a; ISC-V-0021342, n/a; ISC-V-0021341, MN811193.1. ***P. paradoxa*** **L.:** ISC-V-0021360, n/a; ISC-V-0021363, n/a; ISC-V-0021361, MN811195.1; ISC-V-0021362, n/a; ISC-V-0021415, MN811194.1. ***P. truncata*** **Guss. ex Bertol.:** ISC-V-0021377, MN811198.1; ISC-V-0021384, MN811199.1; ISC-V-0021395, MN811197.1; ISC-V-0021373, MN811196.1.
