# Supplementary material for: Variability in ITS1 and ITS2 sequences of historic herbaria and extant (fresh) Phalaris species (Poaceae)
Source: BMC Plant Biol. 2021 Nov 6;21:515. doi: 10.1186/s12870-021-03284-z (PMC8571858; doi:10.1186/s12870-021-03284-z)
Supplement: Supplementary file 1 — Additional file 1: Supplementary Figure 1. Original uncropped image of gel electrophoresis of 200 ng of genomic DNA in Fig. 1. Where lanes 2 and 3 are fresh tissue P. aquatica (PI 476288) and P. arundinacea (PI 241065) respectively. Remaining lanes (4–9) are from herbarium tissue consisting of P. canarensis (619107), P. brachystachys (ISC-V-0021035), P. paradoxa (ISC-V-0021361), P. coerulescens (ISC-V-0021204), P. canarensis (71229) and P. minor (229774). Lanes 1 and 11 are a DNA size marker (FullRanger DNA ladder 1 kb) while lane 10 is a control lane with no sample loaded. Dotted white line over image illustrate where the image was cropped to form Fig. 1. [file 12870_2021_3284_MOESM1_ESM.pptx]

## Slide 1
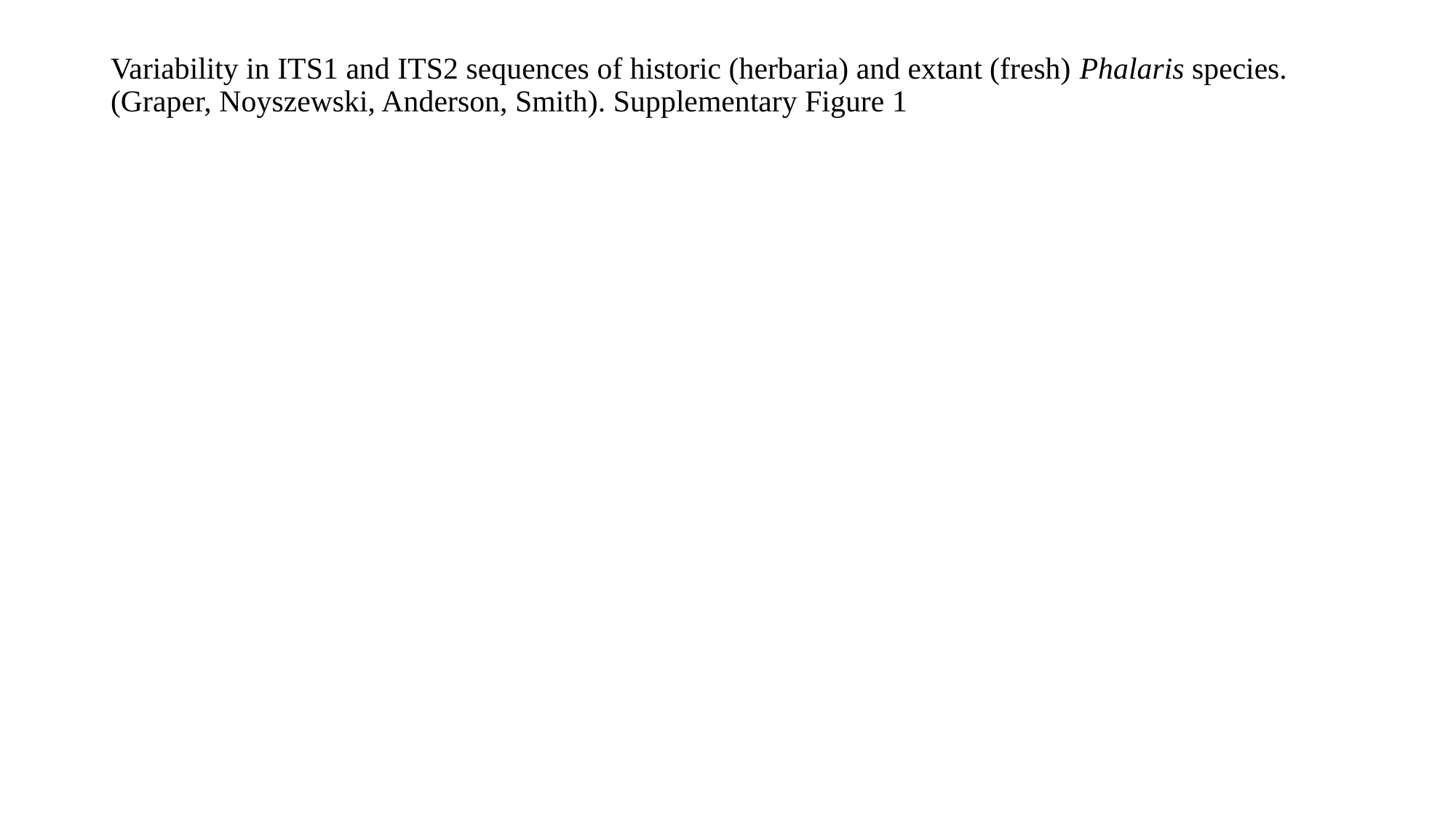

Variability in ITS1 and ITS2 sequences of historic (herbaria) and extant (fresh) Phalaris species. (Graper, Noyszewski, Anderson, Smith). Supplementary Figure 1

## Slide 2
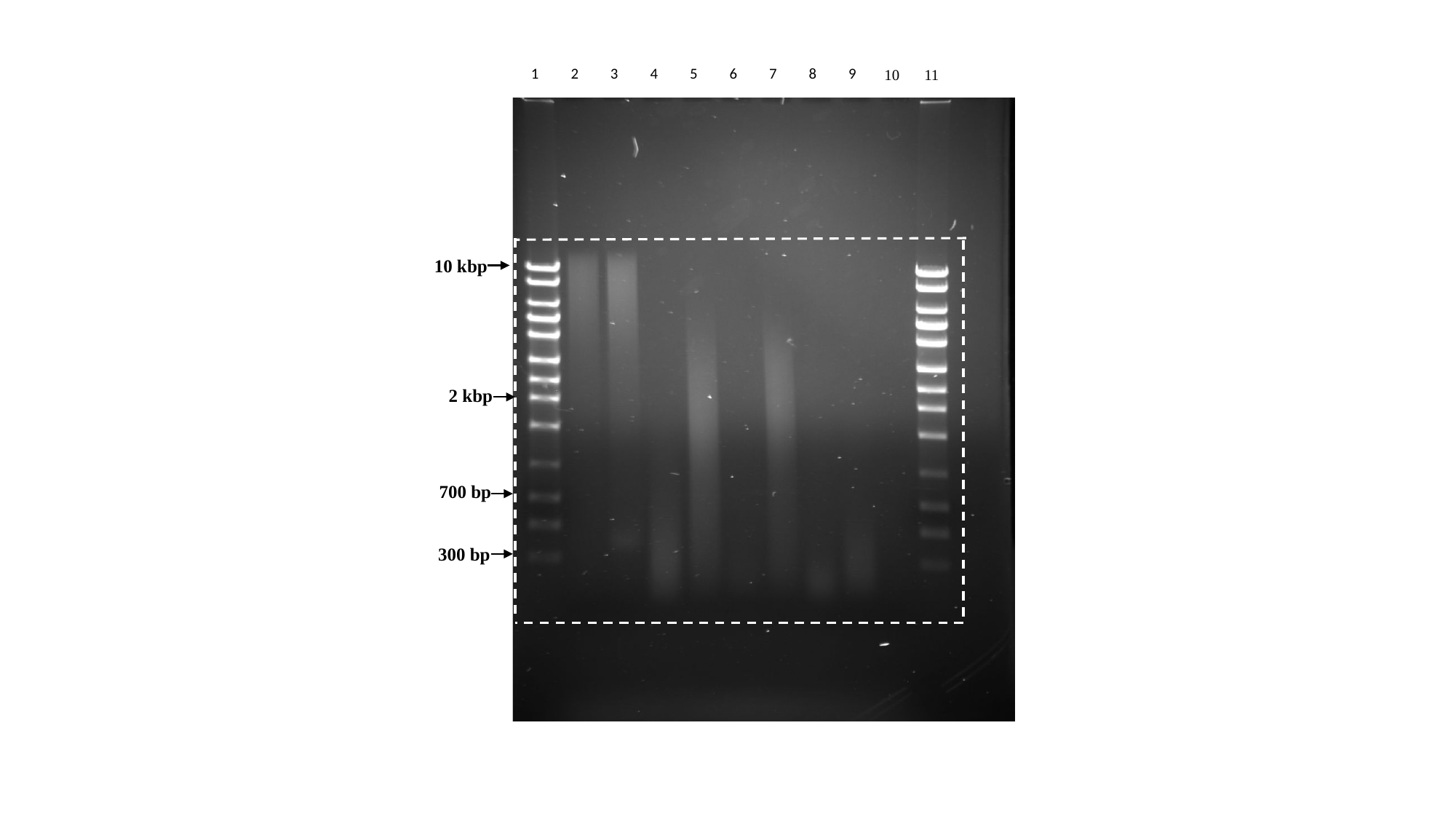

| 1 | 2 | 3 | 4 | 5 | 6 | 7 | 8 | 9 | 10 | 11 |
| --- | --- | --- | --- | --- | --- | --- | --- | --- | --- | --- |
10 kbp
2 kbp
700 bp
300 bp
