# Supplementary material for: Variability in ITS1 and ITS2 sequences of historic herbaria and extant (fresh) Phalaris species (Poaceae)
Source: BMC Plant Biol. 2021 Nov 6;21:515. doi: 10.1186/s12870-021-03284-z (PMC8571858; doi:10.1186/s12870-021-03284-z)
Supplement: Supplementary file 2 — Additional file 2: Supplementary Figure 2. Original uncropped image of gel electrophoresis of PCR amplification examples of the plant specific ITS region in Fig. 3 [51] where panel labels (a, b, c) correspond. Panel a consist of fresh P. canarensis in lane 2 (53.6 ng; PI 578800), P. in lane 3 (57.8 ng; PI 578798), P. aquatica in lane 4 (60.7 ng; PI 476287), P. aquatica in lane 5 (80.7 ng; PI 476288), P. aquatica in lane 6 (59.9 ng; PI 303825), P. arundinacea in lane 7 (52.2 ng; PI 241065), P. arundinacea in lane 8 (28.5 ng/μl; PI 422030), P. canarensis in lane 11 (20 ng; PI 578800), P. canarensis in lane 12 (20 ng; PI 578798), P. aquatica in lane 13 (20 ng; PI 476287), P. aquatica in lane 14 (20 ng; PI 476288), P. aquatica in lane 15 (20 ng; PI 303825), P. arundinacea in lane 16 (20 ng; PI 241065) and P. arundinacea in lane 17 (20 ng; PI 422030). Panel b consists of herbarium specimens of uniform 50 ng quantity where lane 21 is P. canarensis (71226), lane 22 is P. californica (ISC-V-0021043), lane 23 is P. californica (ISC-V-0021040), lane 24 is P. caroliniana (ISC-V-0021097), lane 25 is P. paradoxa (ISC-V-0021360), lane 26 is P. coerulescens (ISC-V-0021199) and lane 27 is P. minor (ISC-V-0021338). Panel c contains PCR re-amplification results of 1/50 dilutions of purified PCR reactions with P. canarensis in lane 31 (484712), P. californica in lane 32 (ISC-V-0021043), P. californica in lane 33 (ISC-V-0021040), P. caroliniana in lane 34 (ISC-V-0021081), P. truncata in lane 35 (ISC-V-0021373), P. truncata in lane 36 (ISC-V-0021384) and P. paradoxa in lane 37 (ISC-V-0021361). Lanes 9, 18, 28 and 38 are control lanes with no sample loaded. Lanes 1, 10, 19, 20, 29, 30 and 39 are a DNA size marker (FullRanger DNA ladder 100 bases). Dotted white line over image illustrate where the image was cropped to form Fig. 3. [file 12870_2021_3284_MOESM2_ESM.pptx]

## Slide 1
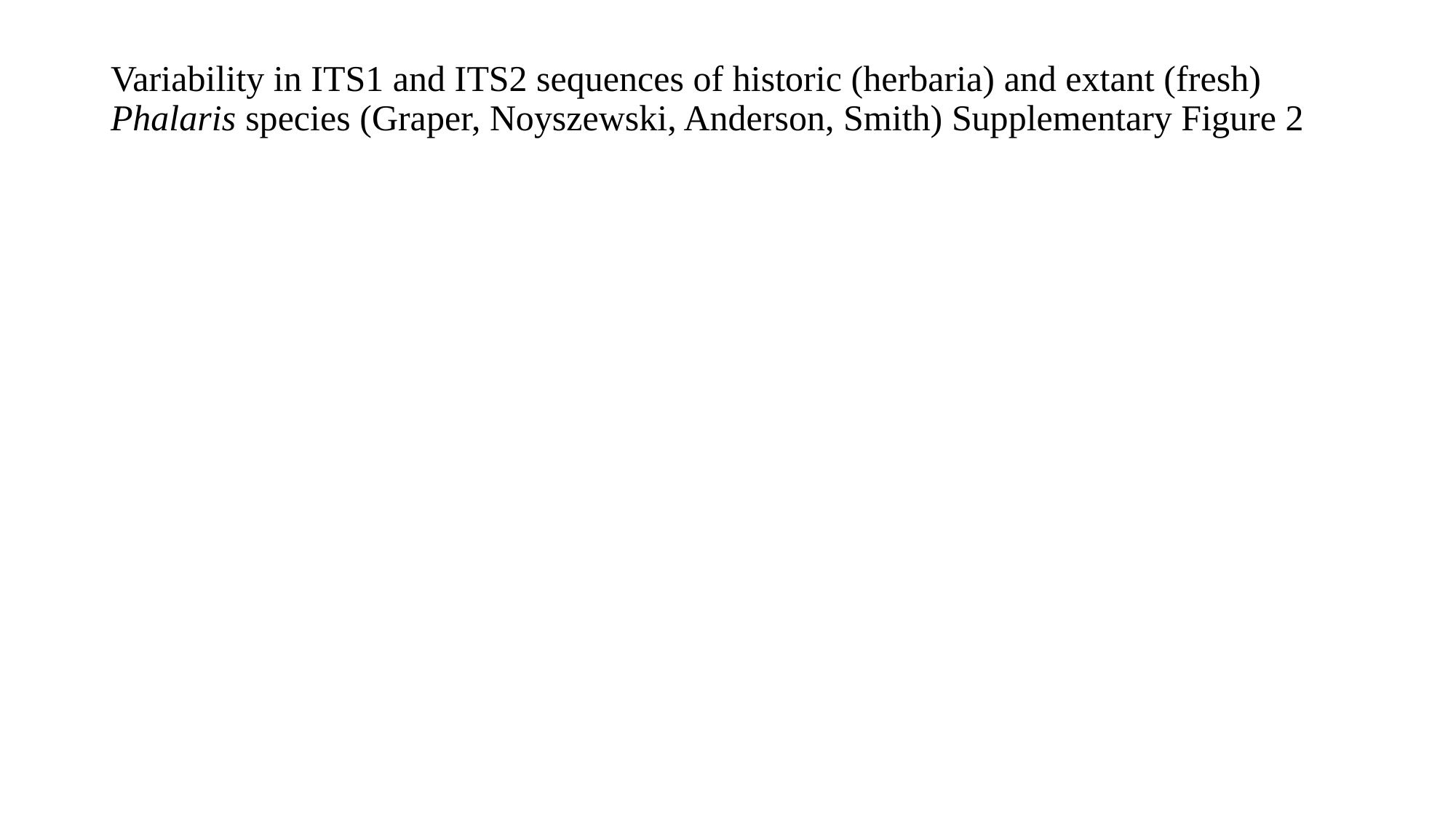

Variability in ITS1 and ITS2 sequences of historic (herbaria) and extant (fresh) Phalaris species (Graper, Noyszewski, Anderson, Smith) Supplementary Figure 2

## Slide 2
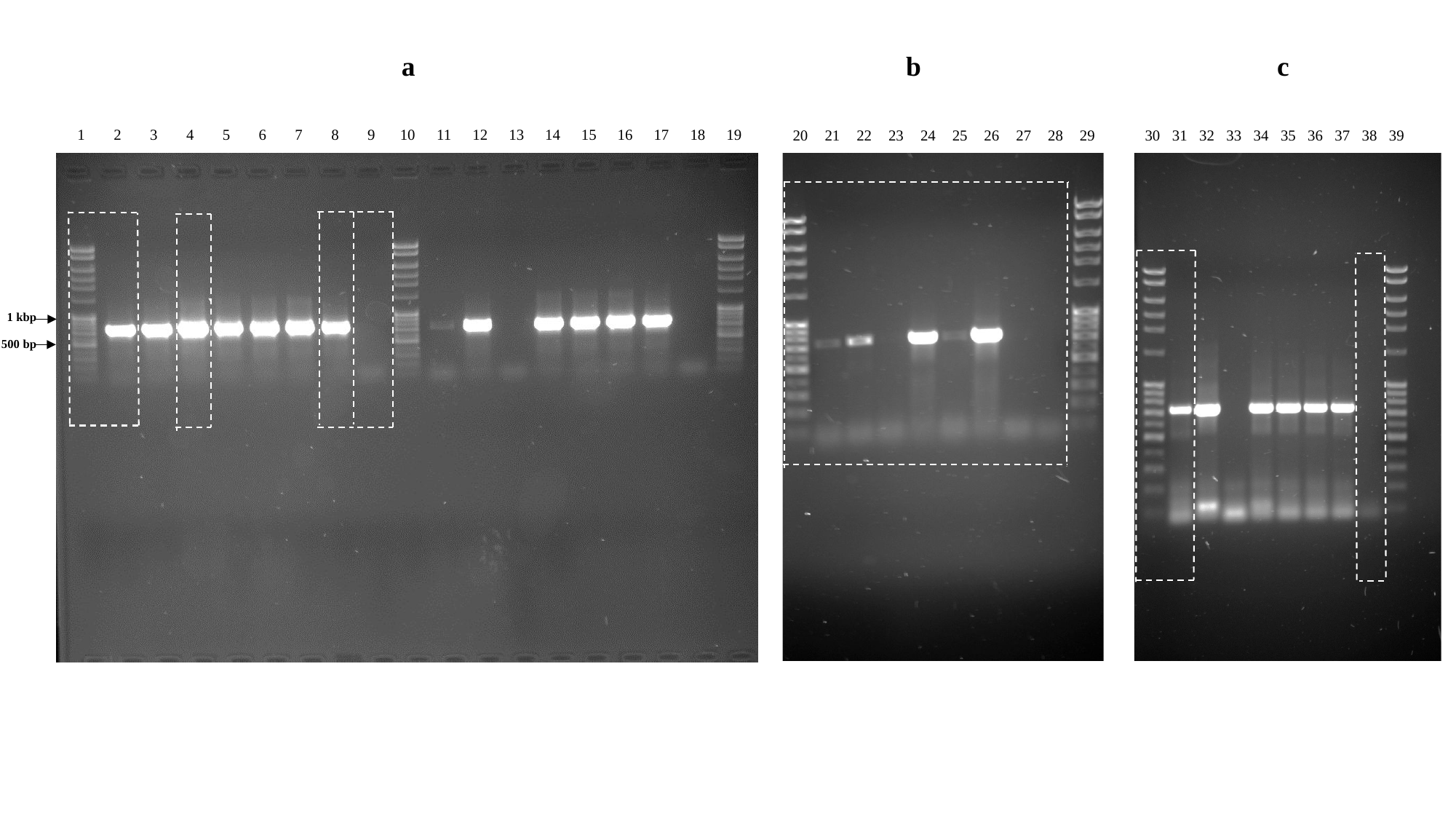

a
b
c
| 20 | 21 | 22 | 23 | 24 | 25 | 26 | 27 | 28 | 29 |
| --- | --- | --- | --- | --- | --- | --- | --- | --- | --- |
| 30 | 31 | 32 | 33 | 34 | 35 | 36 | 37 | 38 | 39 |
| --- | --- | --- | --- | --- | --- | --- | --- | --- | --- |
| 1 | 2 | 3 | 4 | 5 | 6 | 7 | 8 | 9 | 10 | 11 | 12 | 13 | 14 | 15 | 16 | 17 | 18 | 19 |
| --- | --- | --- | --- | --- | --- | --- | --- | --- | --- | --- | --- | --- | --- | --- | --- | --- | --- | --- |
1 kbp
500 bp
