# Supplementary material for: Variability in ITS1 and ITS2 sequences of historic herbaria and extant (fresh) Phalaris species (Poaceae)
Source: BMC Plant Biol. 2021 Nov 6;21:515. doi: 10.1186/s12870-021-03284-z (PMC8571858; doi:10.1186/s12870-021-03284-z)
Supplement: Supplementary file 3 — Additional file 3: Supplementary Figure 3. Sequence alignment used for the distance tree in Fig. 4 with indication of specific single nucleotide polymorphism for Phalaris species. [file 12870_2021_3284_MOESM3_ESM.pptx]

## Slide 1
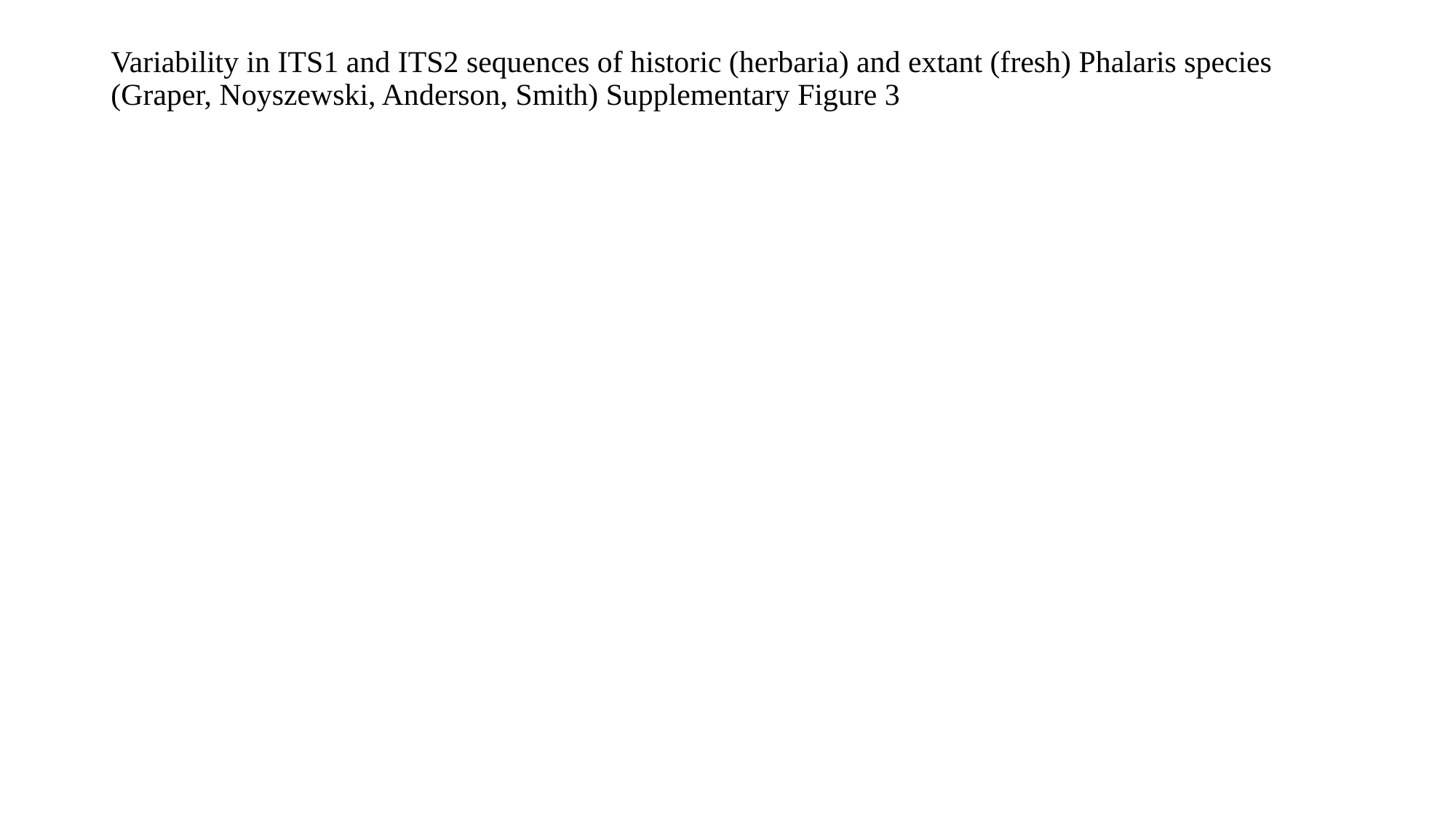

# Variability in ITS1 and ITS2 sequences of historic (herbaria) and extant (fresh) Phalaris species (Graper, Noyszewski, Anderson, Smith) Supplementary Figure 3

## Slide 2
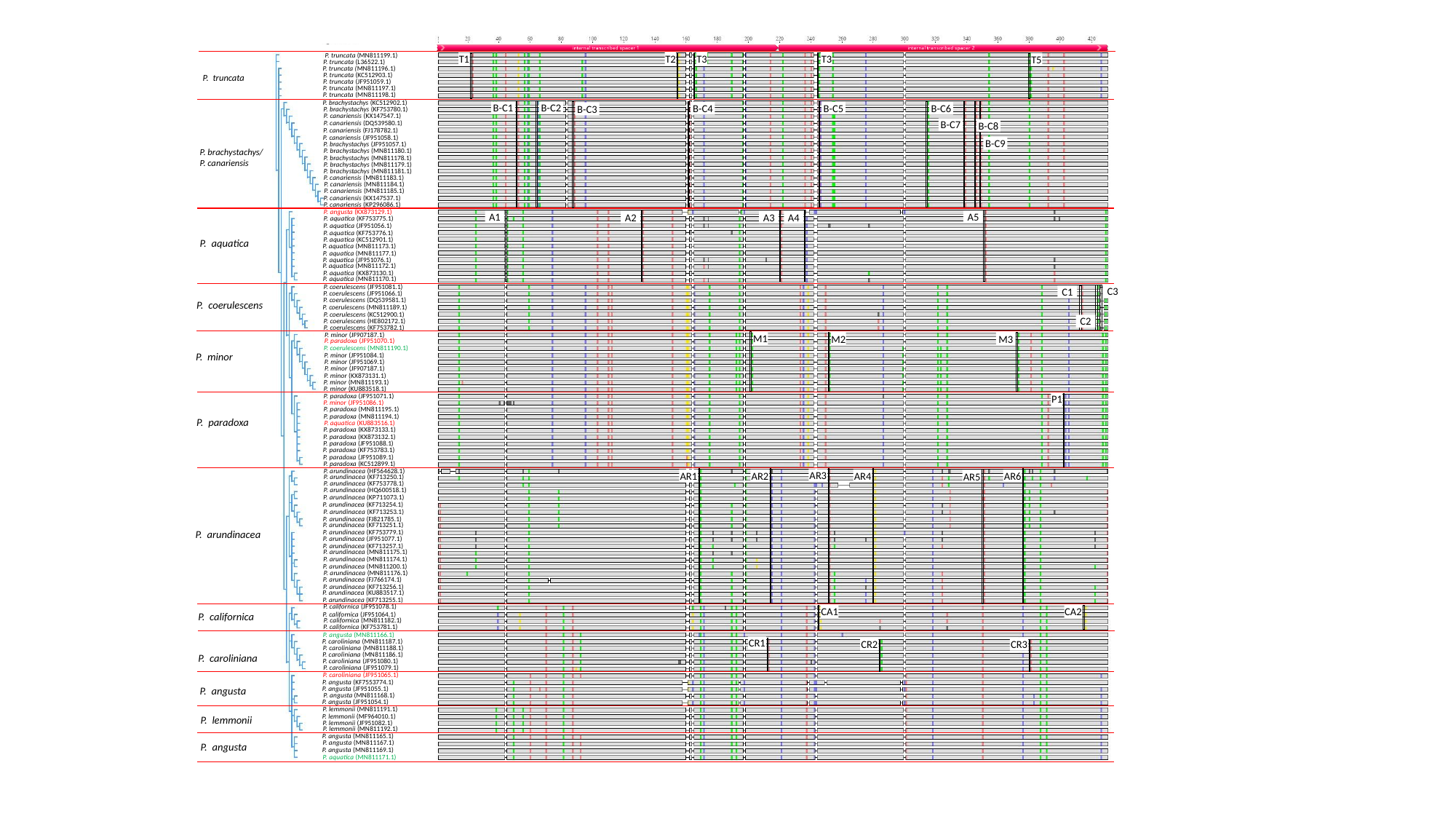

P. truncata (MN811199.1)
T3
T2
T3
T1
T5
P. truncata (L36522.1)
P. truncata (MN811196.1)
P. truncata
P. truncata (KC512903.1)
P. truncata (JF951059.1)
P. truncata (MN811197.1)
P. truncata (MN811198.1)
P. brachystachys (KC512902.1)
B-C1
B-C2
B-C5
B-C9
B-C7
B-C4
B-C6
B-C3
P. brachystachys (KF753780.1)
P. canariensis (KX147547.1)
P. canariensis (DQ539580.1)
B-C8
P. canariensis (FJ178782.1)
P. canariensis (JF951058.1)
P. brachystachys (JF951057.1)
P. brachystachys/
P. canariensis
P. brachystachys (MN811180.1)
P. brachystachys (MN811178.1)
P. brachystachys (MN811179.1)
P. brachystachys (MN811181.1)
P. canariensis (MN811183.1)
P. canariensis (MN811184.1)
P. canariensis (MN811185.1)
P. canariensis (KX147537.1)
P. canariensis (KP296086.1)
P. angusta (KX873129.1)
A1
A5
A3
A2
A4
P. aquatica (KF753775.1)
P. aquatica (JF951056.1)
P. aquatica (KF753776.1)
P. aquatica
P. aquatica (KC512901.1)
P. aquatica (MN811173.1)
P. aquatica (MN811177.1)
P. aquatica (JF951076.1)
P. aquatica (MN811172.1)
P. aquatica (KX873130.1)
P. aquatica (MN811170.1)
P. coerulescens (JF951081.1)
C3
C2
C1
P. coerulescens (JF951066.1)
P. coerulescens
P. coerulescens (DQ539581.1)
P. coerulescens (MN811189.1)
P. coerulescens (KC512900.1)
P. coerulescens (HE802172.1)
P. coerulescens (KF753782.1)
P. minor (JF907187.1)
M1
M2
M3
P. paradoxa (JF951070.1)
P. coerulescens (MN811190.1)
P. minor
P. minor (JF951084.1)
P. minor (JF951069.1)
P. minor (JF907187.1)
P. minor (KX873131.1)
P. minor (MN811193.1)
P. minor (KU883518.1)
P. paradoxa (JF951071.1)
P1
P. minor (JF951086.1)
P. paradoxa (MN811195.1)
P. paradoxa
P. paradoxa (MN811194.1)
P. aquatica (KU883516.1)
P. paradoxa (KX873133.1)
P. paradoxa (KX873132.1)
P. paradoxa (JF951088.1)
P. paradoxa (KF753783.1)
P. paradoxa (JF951089.1)
P. paradoxa (KC512899.1)
P. arundinacea (HF564628.1)
AR3
AR1
AR2
AR4
AR6
AR5
P. arundinacea (KF713250.1)
P. arundinacea (KF753778.1)
P. arundinacea (HQ600518.1)
P. arundinacea (KP711073.1)
P. arundinacea (KF713254.1)
P. arundinacea (KF713253.1)
P. arundinacea (FJ821785.1)
P. arundinacea (KF713251.1)
P. arundinacea
P. arundinacea (KF753779.1)
P. arundinacea (JF951077.1)
P. arundinacea (KF713257.1)
P. arundinacea (MN811175.1)
P. arundinacea (MN811174.1)
P. arundinacea (MN811200.1)
P. arundinacea (MN811176.1)
P. arundinacea (FJ766174.1)
P. arundinacea (KF713256.1)
P. arundinacea (KU883517.1)
P. arundinacea (KF713255.1)
P. californica (JF951078.1)
P. californica
CA1
CA2
P. californica (JF951064.1)
P. californica (MN811182.1)
P. californica (KF753781.1)
P. angusta (MN811166.1)
CR1
P. caroliniana (MN811187.1)
CR2
CR3
P. caroliniana (MN811188.1)
P. caroliniana
P. caroliniana (MN811186.1)
P. caroliniana (JF951080.1)
P. caroliniana (JF951079.1)
P. caroliniana (JF951065.1)
P. angusta (KF7553774.1)
P. angusta
P. angusta (JF951055.1)
P. angusta (MN811168.1)
P. angusta (JF951054.1)
P. lemmonii (MN811191.1)
P. lemmonii
P. lemmonii (MF964010.1)
P. lemmonii (JF951082.1)
P. lemmonii (MN811192.1)
P. angusta (MN811165.1)
P. angusta
P. angusta (MN811167.1)
P. angusta (MN811169.1)
P. aquatica (MN811171.1)
