# Supplementary material for: Variability in ITS1 and ITS2 sequences of historic herbaria and extant (fresh) Phalaris species (Poaceae)
Source: BMC Plant Biol. 2021 Nov 6;21:515. doi: 10.1186/s12870-021-03284-z (PMC8571858; doi:10.1186/s12870-021-03284-z)
Supplement: Supplementary file 4 — Additional file 4: Supplementary Figure 4. Contrast between fresh P. arundinacea (PI 422030; MN811174.1) in panel a with a historic herbarium specimen of P. arundinacea (753,216; MN811176.1) from the University of Minnesota Herbarium in panel b. [file 12870_2021_3284_MOESM4_ESM.pptx]

## Slide 1
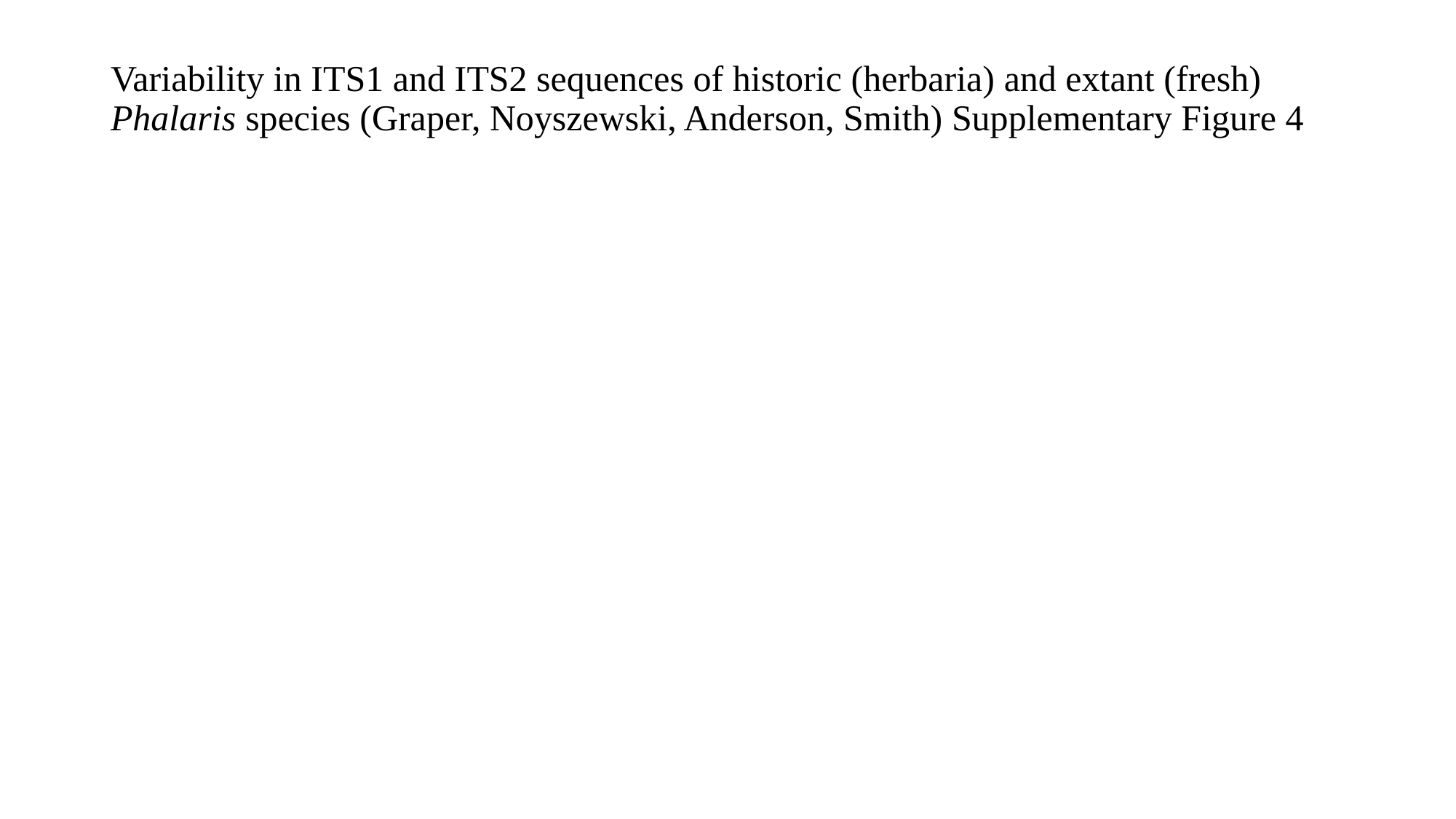

Variability in ITS1 and ITS2 sequences of historic (herbaria) and extant (fresh) Phalaris species (Graper, Noyszewski, Anderson, Smith) Supplementary Figure 4

## Slide 2
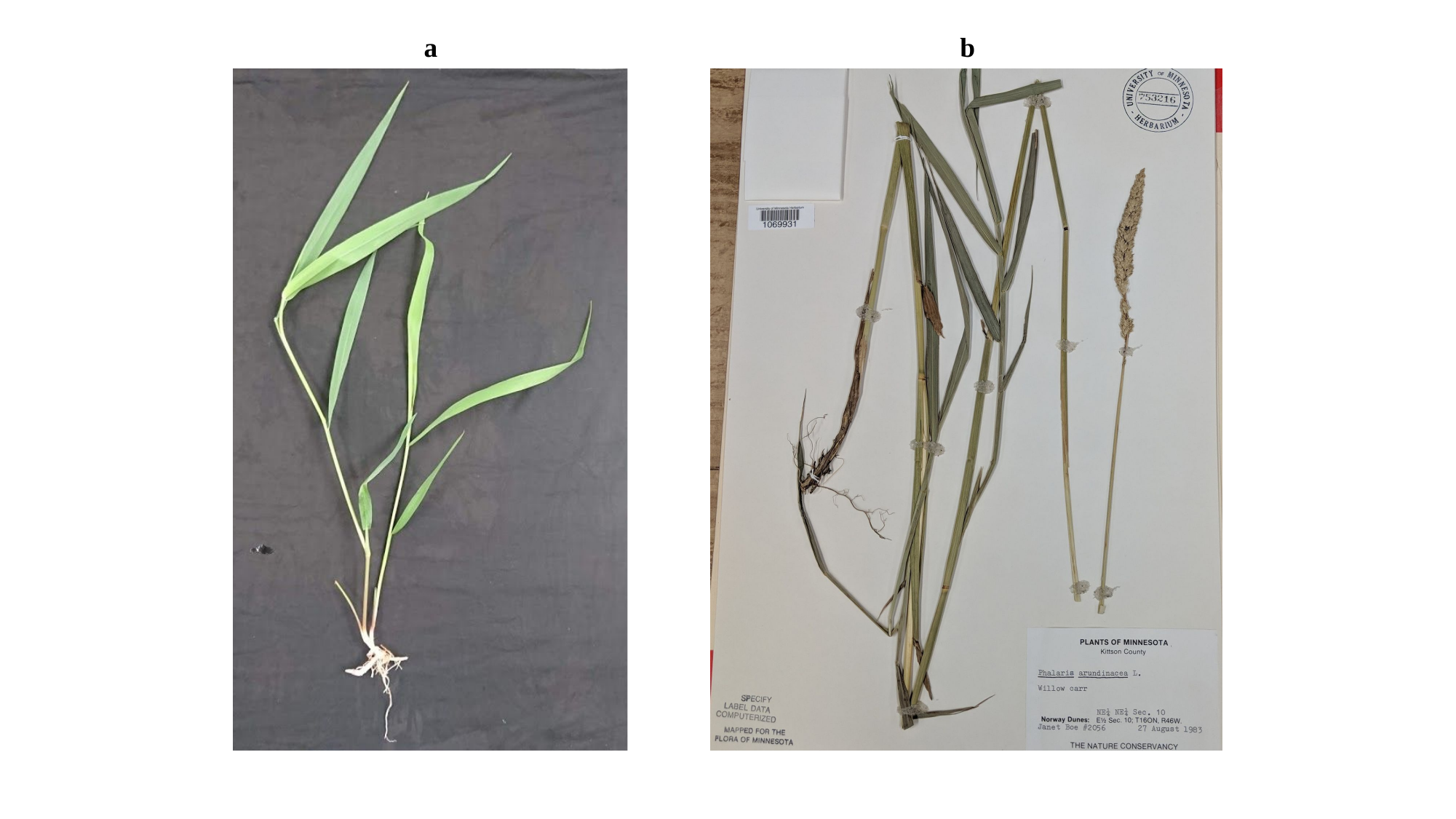

a
b
